# Exogenous adenosine counteracts tigecycline resistance in *tet*(X3)-harboring *Escherichia coli*

**DOI:** 10.1128/spectrum.02382-24

**Published:** 2025-07-07

**Authors:** Jing Sun, Yiming Liu, Jiashen Chang, Ying Liu, Luqi Li, Ran Jiang, Yihan Luo, Shuo Yang, Mei Yang, Xinglong Wang, Juan Wang, Xi Xia, Kangkang Guo, Zengqi Yang, Dongyang Ye

**Affiliations:** 1 College of Veterinary Medicine, Northwest A&F University 12469, Yangling, Shaanxi, China; 2 Experimental Animal Center, Northwest A&F University, Yangling, Shaanxi, China; 3 Life Science Research Core Services, Northwest A&F University, Yangling, Shaanxi, China; 4 Key Laboratory of Ruminant Disease Prevention and Control (West), Ministry of Agriculture and Rural Affairs, Yangling, Shaanxi, China; 5 Key Laboratory of Animal-Derived Bacterial Resistance Monitoring (Co-Construction), Ministry of Agriculture and Rural Affairs, Yangling, Shaanxi, China; 6 National Key Laboratory of Veterinary Public Health and Safety, College of Veterinary Medicine, China Agricultural University, Beijing, China; Nanjing Agricultural University, Nanjing, China

**Keywords:** *tet*(X3), tigecycline, adenosine, antimicrobial resistance, metabolomics

## Abstract

The rapid spread of antibiotic resistance poses a global health crisis. Tigecycline is a last-resort antibiotic, but the recent emergence of the plasmid-borne *tet*(X3) gene conferring high-level tigecycline resistance is deeply concerning. Here, we report a metabolomics-guided approach to overcome *tet*(X3)-mediated resistance. Using untargeted metabolomics, we identified adenosine as a key metabolic biomarker associated with *tet*(X3) expression. Remarkably, supplementation with exogenous adenosine was able to restore tigecycline susceptibility in *tet*(X3)-positive *Escherichia coli* both *in vitro* and *in vivo*. Our mechanistic investigations reveal that adenosine enhances the bactericidal effects of tigecycline by inducing oxidative stress, DNA/RNA damage, and cell membrane disruption in resistant bacteria. This study establishes a powerful metabolomics-driven strategy to potentiate antibiotic efficacy against drug-resistant pathogens. The adenosine-based adjuvant therapy represents a promising approach to combat the global crisis of antibiotic resistance.

**IMPORTANCE:**

The emergence and widespread dissemination of the high-level tigecycline resistance gene *tet*(X3) have posed a significant challenge to the efficacy of tigecycline, which serves as the “last line of defense” against antimicrobial-resistant bacteria. Although tigecycline has not been approved for veterinary clinical use, constant detection of *tet*(X3) genes and new subtypes in livestock farming environments poses a substantial threat to public health safety. While developing novel antibiotics is an effective approach to eradicate resistance genes/bacteria, it entails considerable costs and a lengthy timeframe. This study discovered that exogenous adenosine can effectively restore the sensitivity of *tet*(X3)-positive *Escherichia coli* to tigecycline through metabolic reprogramming based on a non-targeted metabolomics strategy. The findings are highly significant for exploring comprehensive mechanisms underlying bacterial multidrug resistance, utilizing metabolic reprogramming strategies to curb the spread of novel resistant genes, and treating clinical infections caused by *tet*(X3)-positive bacteria.

## INTRODUCTION

Since the serendipitous discovery of penicillin by Alexander Fleming in 1928 and its subsequent application in clinical practice, antimicrobial agents have played a pivotal role in both human and animal health. These medications effectively combat a wide range of infectious diseases through their potent antibacterial effects, thereby safeguarding the well-being and preserving lives (1, 2). However, the extensive and inappropriate use of antimicrobial drugs has significantly contributed to the emergence and spread of resistant strains, encompassing multidrug-resistant and extensively drug-resistant variants along with “superbugs” (3). Consequently, traditional anti-infective treatments have become progressively less effective due to the gradual rise in antibiotic resistance (4). The escalating crisis of resistance presents unprecedented challenges and risks not only in the field of human medicine for treating bacterial infections, but also within veterinary practices and public health safety (5).

Tigecycline (TGC), a glycylcycline antibiotic and the third generation of tetracycline (TET) antibiotics, has garnered significant attention due to its distinctive antibacterial mechanism and exceptional efficacy. In comparison to conventional TET drugs, TGC exhibits enhanced binding affinity with the ribosomal subunit 30S and remains unaffected by the primary resistance mechanisms associated with TET drugs, such as efflux pumps, ribosomal protection proteins, enzymatic modifications, and ribosomal mutations (6). Therefore, it offers innovative prospects for addressing bacterial resistance issues in anti-infection treatment and is regarded as the ultimate therapeutic option subsequent to carbapenems and glycopeptides, serving as the final line of defense (7, 8). The mechanisms underlying antibiotic resistance are diverse and complex, often involving genetic mutations and the acquisition of resistance genes through horizontal gene transfer. Among these resistance mechanisms, tetracycline resistance genes, particularly *tet*(X3), have emerged as significant contributors to the spread of antimicrobial resistance (9). Unlike traditional tetracycline resistance genes that typically confer resistance through efflux or ribosomal protection, *tet*(X3) provides a unique and potent resistance strategy by directly destroying the antibiotic molecule, encoding the Tet(X3) enzyme, which confers resistance to TGC and other TETs by enzymatically degrading these antibiotics in the presence of FAD, NADPH, O_2_, and Mg^2+^ (10, 11). This gene has been increasingly detected in various bacterial species, including clinically important pathogens such as *Escherichia coli* and *Klebsiella pneumoniae*. The rapid spread of *tet*(X3) is particularly concerning due to its ability to confer resistance to newer generations of tetracycline antibiotics, including tigecycline, which has been a last-line treatment option for multidrug-resistant infections. Understanding the molecular mechanisms, transmission dynamics, and epidemiological patterns of *tet*(X3) is crucial for developing effective strategies to mitigate its spread and preserve antibiotic efficacy. Despite the efficacy of developing innovative antibiotics in combating resistant genes and bacteria, this approach incurs substantial costs and necessitates a significant time investment from drug discovery to approval process, which can extend up to 15–20 years (3).

Traditionally, the predominant focus of studies on bacterial drug resistance has been centered around the analysis of drug targets, efflux pumps, enzyme activity, and other associated factors (5). While this approach has yielded significant advancements, it has also resulted in an oversimplified perspective that attributes drug resistance solely to the inhibition of drug targets or mechanisms such as hydrolysis or efflux (12, 13). Recently, there has been a scholarly shift toward investigating metabolic regulation, leading to the discovery of a significant correlation between bacterial metabolic state and their drug resistance phenotype (14, 15). The elucidation of metabolic regulatory mechanisms in resistant bacteria and the identification of metabolites capable of reversing resistance through metabolic reprogramming bear immense scientific significance for gaining a completely novel perspective on the mechanism of bacterial drug resistance, as well as fostering the development of innovative antibacterial enhancers. We have previously demonstrated that metabolic regulation plays a pivotal role in mediating resistance to varying concentrations of antibiotics, implying a critical involvement of metabolic reprogramming in bacterial resistance and antibiotic efficacy (16). Considering the pivotal roles of FAD, NADPH, and other metabolites in cellular metabolism (10, 17), we propose that *tet*(X3)-mediated resistance is intricately intertwined with the metabolic state of bacteria. Furthermore, we hypothesize that manipulation of metabolic reprogramming can effectively modulate the *tet*(X3)-mediated resistance pathway, potentially leading to reversal of resistance.

In this study, we employed ultra-high-performance liquid chromatography tandem mass spectrometry-based untargeted metabolomics to investigate the metabolic regulatory mechanism underlying resistance mediated by the *tet*(X3) gene. Through strategic metabolic reprogramming, we identified adenosine (Ado) with potential for reversing TGC resistance in *tet*(X3) gene-positive bacteria and elucidated their underlying mechanisms and potential applications in overcoming drug resistance. Our research holds significant scientific and practical value in unraveling novel resistance mechanisms of the *tet*(X3) gene, discovering new compounds for combating drug resistance, proposing innovative strategies for controlling the *tet*(X3) gene, and uncovering novel antibacterial synergists.

## RESULTS

### Reprogramming of metabolic profiles in *E. coli* carrying the *tet*(X3) gene

**Fig 1:**
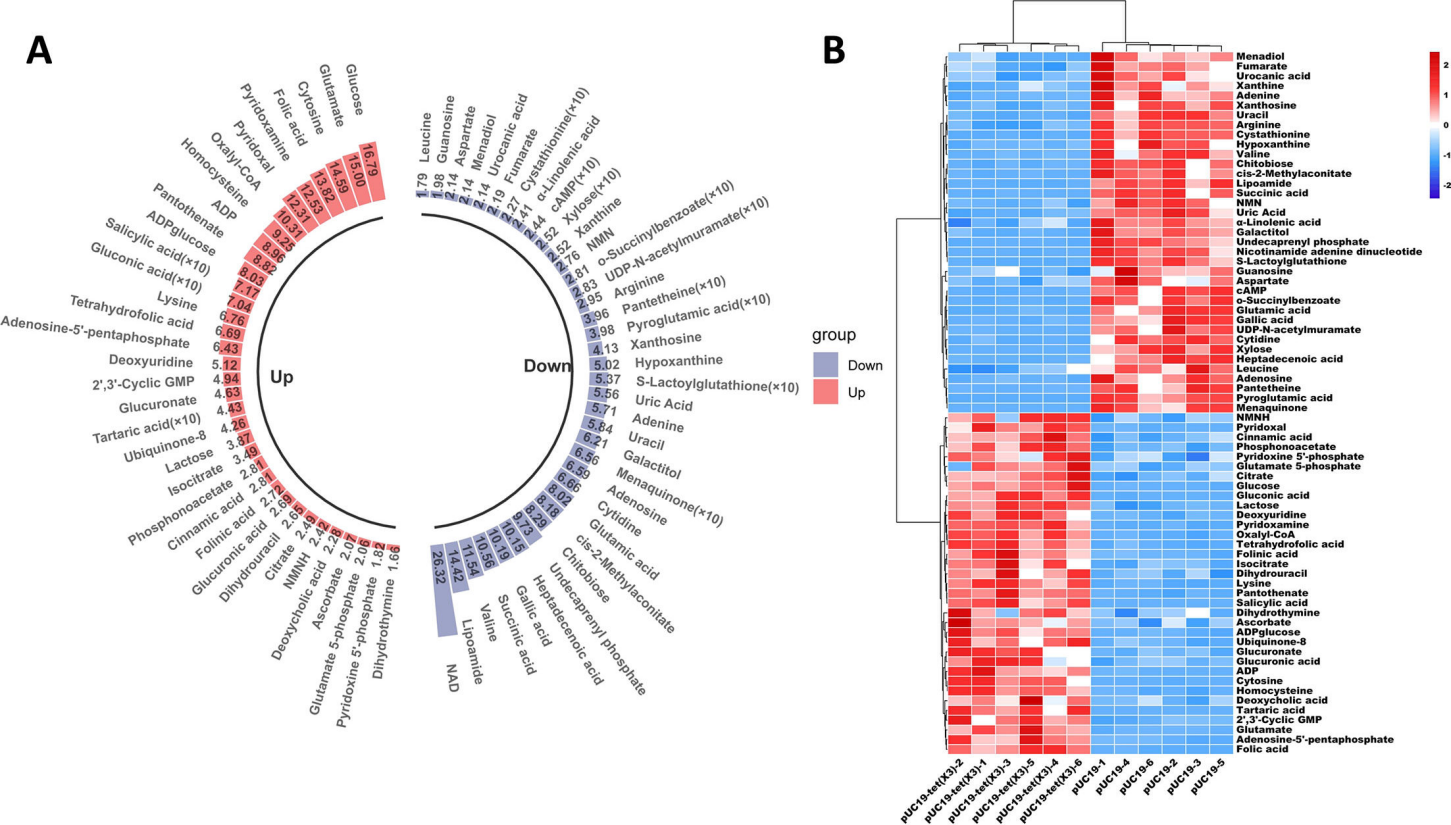
Reprogramming of metabolic profiles in *E. coli* carrying the *tet*(X3) gene. (**A**) Upregulation and downregulation of differential metabolites in *tet*(X3) gene-positive *E. coli*. A total of 72 differential metabolites were identified, with 35 upregulated and 37 downregulated in the *tet*(X3)-carrying strain. The upregulated metabolites primarily consist of pantothenate, ADP, cytosine, glucose, folic acid, and glutamate, while the downregulated metabolites mainly include gallic acid, valine, NAD, and succinic acid. (**B**) Heatmap analysis of differential metabolite spectrum in *tet(*X3) gene-positive *E. coli*. The heatmap showcases the fluctuations of multiple metabolites, with each column representing a specific treatment condition, while each row corresponds to a distinct metabolite. After undergoing normalization, the spectrum of metabolite changes extends from −2 (blue) to 2 (red). The dendrogram positioned at the top showcases the clustering relationship among samples, revealing that specific treatment conditions exhibit a greater resemblance in terms of metabolic patterns.

We established a research model using engineered strains of *E. coli* DH5α (pUC19/*tet*(X3)) and *E. coli* DH5α (pUC19), followed by employing metabolomics techniques based on ultra-high-performance liquid chromatography-hybrid quadrupole-Orbitrap mass spectrometry (UHPLC-QE-Orbitrap-MS) to conduct a comprehensive analysis of the impact of *tet*(X3) gene expression on the metabolic profile of *E. coli*. By applying advanced data processing methods, such as orthogonal partial least squares-discriminant analysis, significant metabolic differences between *E. coli* DH5α (pUC19*/tet*(X3)) and control strains were revealed. Specifically, we identified 72 differential metabolites, with 35 upregulated and 37 downregulated in the *tet*(X3)-carrying strain (Fig. 1A and Table S1). The upregulated metabolites primarily consist of pantothenate, ADP, cytosine, glucose, folic acid, and glutamate. Meanwhile, the downregulated metabolites mainly include gallic acid, valine, NAD, and succinic acid (Fig. 1A). The upregulated metabolites are predominantly concentrated in carbohydrates and certain nucleotide-related substances. On the other hand, the downregulated metabolites encompass various amino acids and purine/pyrimidine derivatives (Fig. 1A and Table S1). It is worth noting that some values for these metabolites are denoted as “(×10),” indicating a multiplication factor of 10 should be applied to obtain their actual values (Fig. 1A). These findings reflect a significant enhancement in carbohydrate metabolism and specific nucleotide metabolism in *tet*(X3)-positive bacteria, while amino acid metabolism and certain purine/pyrimidine metabolism are attenuated.

The metabolomic analysis of *E. coli* strains carrying the *tet*(X3) gene and sensitive strains was conducted using a heatmap clustering approach (Fig. 1B). The results unequivocally demonstrate the substantial impact of *tet*(X3) gene expression on the metabolome of *E. coli*. The distinct categorization of the six samples comprising *E. coli* DH5α (pUC19/*tet*(X3)) and *E. coli* DH5α (pUC19) reflects the profound metabolic alterations induced by the presence of the *tet*(X3) gene. The heatmap showcases the fluctuations of multiple metabolites, with each column representing a specific treatment condition, while each row corresponds to a distinct metabolite. After undergoing normalization, the spectrum of metabolite changes extends from −2 (blue) to 2 (red). The dendrogram positioned at the top showcases the clustering relationship among samples, revealing that specific treatment conditions exhibit a greater resemblance in terms of metabolic patterns. The samples of *E. coli* DH5α (pUC19/*tet*(X3)) and *E. coli* DH5α (pUC19) clearly form two distinct clusters, indicating their differential metabolic profiles. The observed upregulation and downregulation patterns in the heatmap are consistent with Fig. 1A, suggesting significant impacts on specific metabolic pathways such as amino acid metabolism and nucleotide metabolism, among others. Importantly, comparative metabolomic analysis revealed significant differences in the abundance of multiple metabolites between *E. coli* DH5α (pUC19/*tet*(X3)) and *E. coli* DH5α (pUC19) (Fig. 1B), suggesting a systematic perturbation of bacterial metabolism induced by the presence of the *tet*(X3) gene.

### The *tet*(X3) gene orchestrates modifications in metabolic pathways

**Fig 2:**
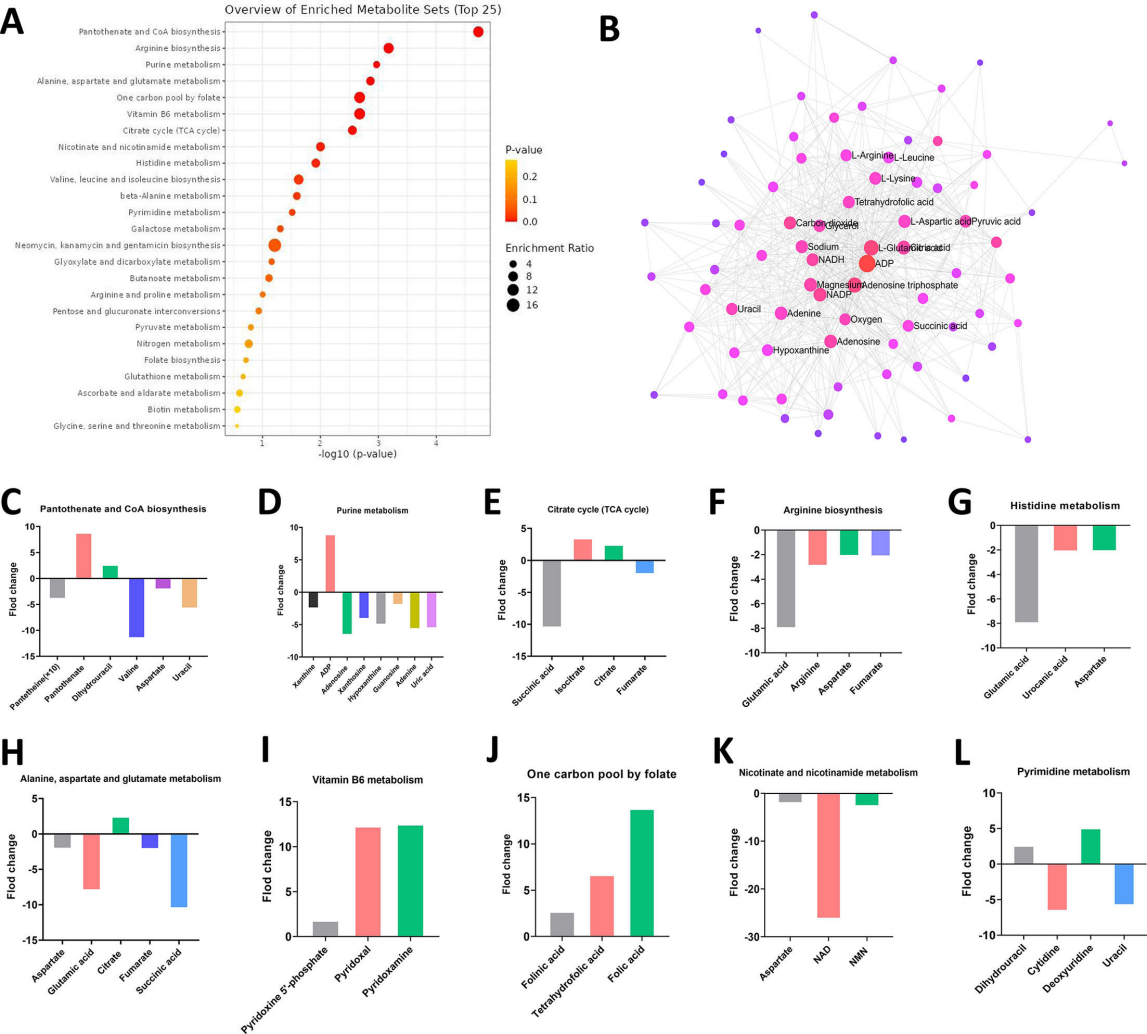
The *tet*(X3) gene orchestrates modifications in metabolic pathways. (**A**) Metabolic pathway enrichment analysis. (**B**) Metabolite and metabolite interaction network analysis. (**C**) Metabolic perturbations of key metabolites in pantothenate and CoA biosynthesis. (**D**) Metabolic perturbations of key metabolites in purine metabolism. (**E**) Metabolic perturbations of key metabolites in the TCA cycle. (**F**) Metabolic perturbations of key metabolites in arginine biosynthesis. (**G**) Metabolic perturbations of key metabolites in histidine metabolism. (**H**) Metabolic perturbations of key metabolites in alanine, aspartate, and glutamate metabolism. (**I**) Metabolic perturbations of key metabolites in vitamin B6 metabolism. (**J**) Metabolic perturbations of key metabolites in one-carbon pool by folate. (**K**) Metabolic perturbations of key metabolites in nicotinate and nicotinamide metabolism. (**L**) Metabolic perturbations of key metabolites in pyrimidine metabolism.

To further elucidate the potential mechanisms underlying *tet*(X3) gene-mediated metabolic changes, we conducted Kyoto Encyclopedia of Genes and Genomes (KEGG) pathway enrichment analysis on the identified differential metabolites (Fig. 2A). The results revealed that *E. coli* carrying the *tet*(X3) gene exhibited significant alterations in various metabolic pathways compared to *E. coli* DH5α(pUC19), including pantothenate and CoA biosynthesis, arginine biosynthesis, purine metabolism, alanine, aspartate and glutamate metabolism, folate-mediated one-carbon metabolism, vitamin B6 metabolism, tricarboxylic acid cycle, nicotinate and nicotinamide metabolism, histidine metabolism, valine, leucine, and isoleucine biosynthesis, β-alanine metabolism, and pyrimidine metabolism (Fig. 2A). Additionally illustrated in Fig. 2B is a metabolic network analysis highlighting ADP, NADH, and NADP as central entities within this intricate network.

The metabolic profiling data presented in this study reveal substantive shifts across a diverse array of biochemical pathways. In the pantothenate and CoA biosynthesis axis (Fig. 2C), we observed marked reductions in the levels of pantetheine and valine, alongside modest decreases in aspartate and uracil. Conversely, pantothenate and dihydrouracil exhibited noteworthy increases. The purine metabolic network (Fig. 2D) displayed a striking elevation in ADP levels, juxtaposed with varied degrees of decline in metabolites such as xanthine, xanthosine, hypoxanthine, guanosine, adenine, and uric acid. Turning to the citric acid cycle (Fig. 2E), isocitrate and citrate demonstrated moderate increases, while succinic acid underwent a significant reduction. Fumarate levels also showed a slight decrease. In the arginine biosynthesis pathway (Fig. 2F), glutamic acid experienced a notable decline, accompanied by smaller reductions in arginine, aspartate, and fumarate. The histidine metabolism axis (Fig. 2G) exhibited a substantial drop in glutamic acid, along with minor decreases in urocanic acid and aspartate. For alanine, aspartate, and glutamate metabolism (Fig. 2H), citrate was the sole metabolite to exhibit an increase, while succinic acid and glutamic acid displayed the most pronounced reductions. Aspartate and fumarate also showed smaller decreases. In the vitamin B6 pathway (Fig. 2I), pyridoxine 5´-phosphate underwent the smallest increase (1.82-fold), whereas pyridoxal and pyridoxamine both demonstrated marked elevations (12.3-fold and 12.5-fold). The one-carbon pool facilitated by folate showed a significant rise in folic acid, accompanied by notable increases in folinic acid and tetrahydrofolate (Fig. 2J). Nicotinate and nicotinamide metabolism was characterized by a substantial decline in NAD, while nicotinamide mononucleotide and aspartate exhibited a slight decrease (Fig. 2K). Finally, the pyrimidine metabolic branch displayed a pronounced reduction in cytidine and uracil, with dihydrouracil and deoxyuridine also showing modest increases (Fig. 2L). These wide-ranging metabolic alterations suggest a profound reconfiguration of cellular physiology, potentially reflecting adaptive responses to specific experimental conditions or treatments. The coordinated shifts observed across central carbon metabolism, nucleotide synthesis, amino acid pathways, and cofactor systems underscore the complex and interconnected nature of this metabolic reprogramming.

### Ado effectively mitigates TGC resistance in bacteria carrying the *tet*(X3) gene

**Fig 3:**
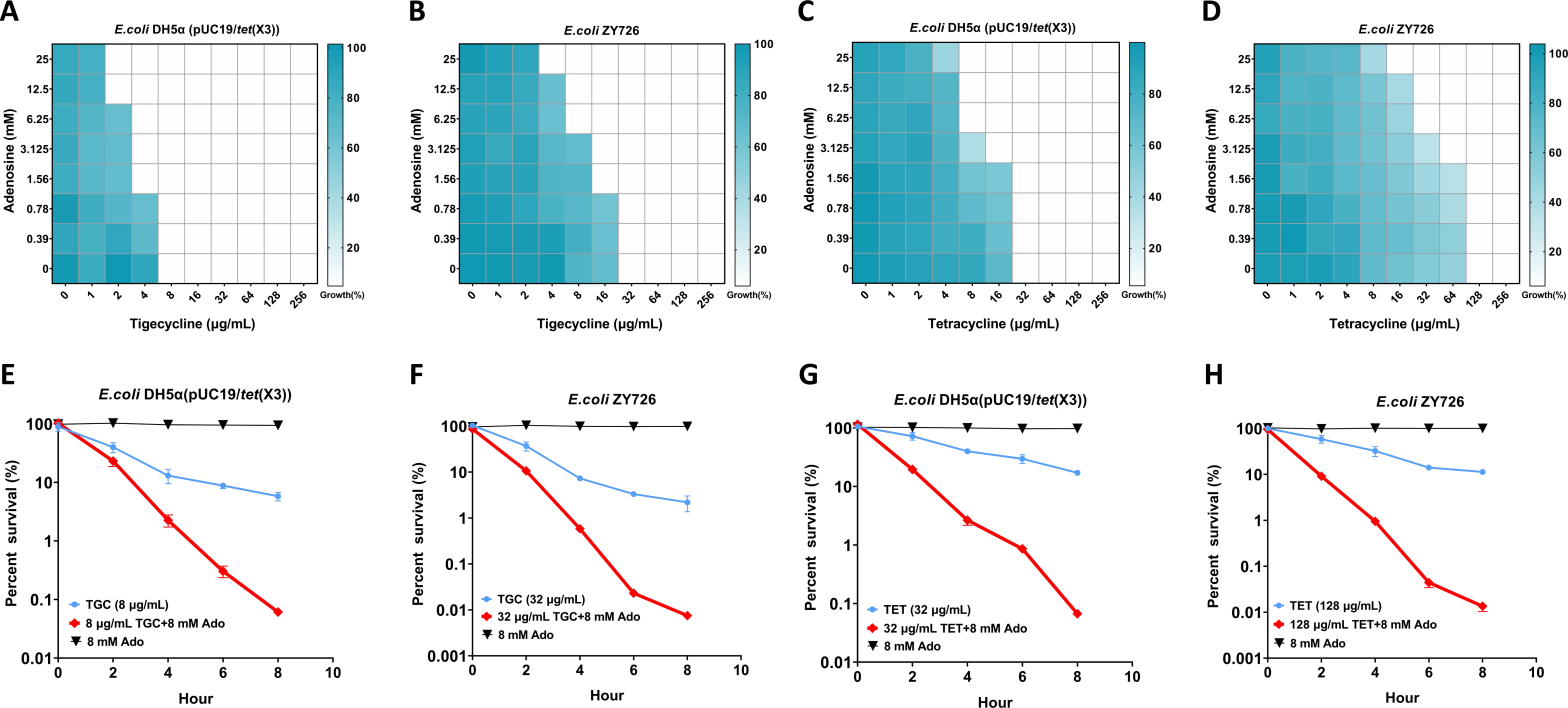
Synergistic antibacterial effect of adenosine in combination with tigecycline or tetracycline. (**A**) The checkerboard method was employed to assess the synergistic antibacterial effect of adenosine in combination with tetracycline on engineered strains. (**B**) The checkerboard method was utilized to evaluate the synergistic antibacterial effect of adenosine in combination with tetracycline on natural *tet*(X3)-carrying strain. (**C**) The checkerboard method was applied to determine the synergistic antibacterial effect of adenosine in combination with doxycycline on engineered strains. (**D**) The checkerboard method was employed to ascertain the synergistic antibacterial effect of adenosine in combination with doxycycline on natural *tet*(X3)-carrying strain. (**E**) Adenosine and tetracycline were combined for generating a synergy killing curve using engineered strains. (**F**) Adenosine and tetracycline were combined for generating a synergy killing curve using natural *tet*(X3)-carrying strain. (**G**) Adenosine and doxycycline were combined for generating a synergy killing curve using engineered strains. (**H**) Adenosine and doxycycline were combined for generating a synergy killing curve using natural *tet*(X3)-carrying strain. (*N* = 3 independent experiments, each performed in duplicate), bars indicate means, and error bars indicate standard deviation.

We aimed to identify potential antibacterial adjuvants capable of reversing or mitigating *tet*(X3)-mediated resistance, thereby enhancing the efficacy of existing antibiotics. Through systematic screening of the differential metabolites identified in this study, we discovered that Ado acts as a potent enhancer of TGC’s antibacterial activity by exhibiting broad-spectrum inhibitory effects. This finding holds promise for combating *tet*(X3)-mediated resistance and may have implications for addressing other forms of antibiotic resistance. We employed the checkerboard broth microdilution method to assess the synergistic effects of Ado combined with two TET antibiotics, TGC and TET (Fig. 3A through D). This method enabled us to systematically test various concentration combinations to accurately determine optimal synergistic conditions. Our study utilized both a *tet*(X3)-positive *E. coli* strain, *E. coli* DH5α (pUC19/*tet*(X3)), and a natural *tet*(X3)-carrying *E. coli* named ZY726. The results demonstrate that Ado synergistically enhances the antibacterial activity of both TGC and TET against both *tet*(X3)-positive engineered strain *E. coli* DH5α (pUC19/*tet*(X3)) and natural *tet*(X3)-carrying strain *E. coli* ZY726. Specifically, for *E. coli* DH5α (pUC19/*tet*(X3)), Ado concentrations ranging from 1.56 mM to 6.25 mM resulted in a 50% reduction in the minimum inhibitory concentration (MIC) of TGC, while Ado concentrations exceeding 12.5 mM resulted in a 75% reduction (fractional inhibitory concentration index [FICI] <0.3124) (Fig. 3A). Similarly, for natural *tet*(X3)-carrying strain *E. coli* ZY726, Ado concentrations exceeding 1.56 mM reduced the MIC of TGC by 50% to 87.5% (FICI <0.1874) (Fig. 3B). Notably, Ado also demonstrated synergy with TET. For *E. coli* DH5α (pUC19/*tet*(X3)), Ado concentrations ≥3.125 mM reduced the MIC of TET by 50% to 75% (FICI <0.375) (Fig. 3C). Similarly, for *E. coli* ZY726, the MIC of TET decreased by 50% to 87.5% with Ado concentrations ≥3.125 mM (FICI <0.25) (Fig. 3D).

### Ado synergistically enhances the bactericidal efficacy of TGC

To further elucidate the synergistic antibacterial effects of Ado and TET antibiotics, we investigated the bacterial growth curve and time-kill kinetics of Ado in combination with TGC and TET against *E. coli*. We monitored the growth dynamics of bacterial strains under the influence of TGC or TET, both alone and in combination with Ado (Fig. 3E through H). Our findings revealed that both natural *tet*(X3)-carrying and engineered strains experienced varying degrees of growth inhibition when exposed to either antibiotic. Notably, the combination of these TET-class antibiotics with Ado resulted in markedly enhanced growth inhibition (Fig. S1A through D). Time-kill kinetics analysis provided further insights into the bactericidal effects of these treatments. For instance, when *E. coli* DH5α (pUC19/*tet*(X3)) was exposed to 8 µg/mL TGC alone, the survival rates were 39.97 ± 6.28%, 13.07 ± 2.89%, 8.84 ± 0.83%, and 5.81 ± 0.80% at 2, 4, 6, and 8 h, respectively. The addition of Ado significantly enhanced TGC’s bactericidal activity, reducing survival rates to 23.18 ± 3.62%, 2.26 ± 0.43%, 0.30 ± 0.06%, and 0.06 ± 0.01% at the corresponding time points (Fig. 3E). Similarly, the natural *tet*(X3)-carrying strain *E. coli* ZY726 exhibited heightened susceptibility to the combination treatment. Under 32 µg/mL TGC alone, survival rates were 36.83 ± 6.55%, 7.31 ± 0.42%, 3.32 ± 0.22%, and 2.19 ± 0.67% at 2, 4, 6, and 8 h, respectively. The addition of Ado markedly reduced these rates to 10.61 ± 0.43%, 0.58 ± 0.04%, 0.023 ± 0.003%, and 0.0075 ± 0.0009% (Fig. 3F). When exposed to 32 µg/mL TET, *E. coli* DH5α (pUC19/*tet*(X3)) demonstrated survival rates of 72.59 ± 8.69%, 40.05 ± 3.38%, 29.81 ± 4.19%, and 17.19 ± 0.98% at 2, 4, 6, and 8 h, respectively. The combination with Ado dramatically enhanced TET’s efficacy, reducing survival rates to 19.61 ± 1.26%, 2.66 ± 0.40%, 0.86 ± 0.11%, and 0.067 ± 0.007% (Fig. 3G). For *E. coli* ZY726, treatment with 128 µg/mL TET resulted in survival rates of 58.55 ± 9.05%, 32.30 ± 6.32%, 14.04 ± 0.91%, and 11.33 ± 0.93% at 2, 4, 6, and 8 h, respectively. The addition of Ado significantly potentiated TET’s bactericidal activity, reducing survival rates to 9.13 ± 0.29%, 0.95 ± 0.09%, 0.0443 ± 0.0083%, and 0.0135 ± 0.0026% (Fig. 3H).

### Co-administration of Ado and TGC leads to intracellular accumulation of ROS

**Fig 4:**
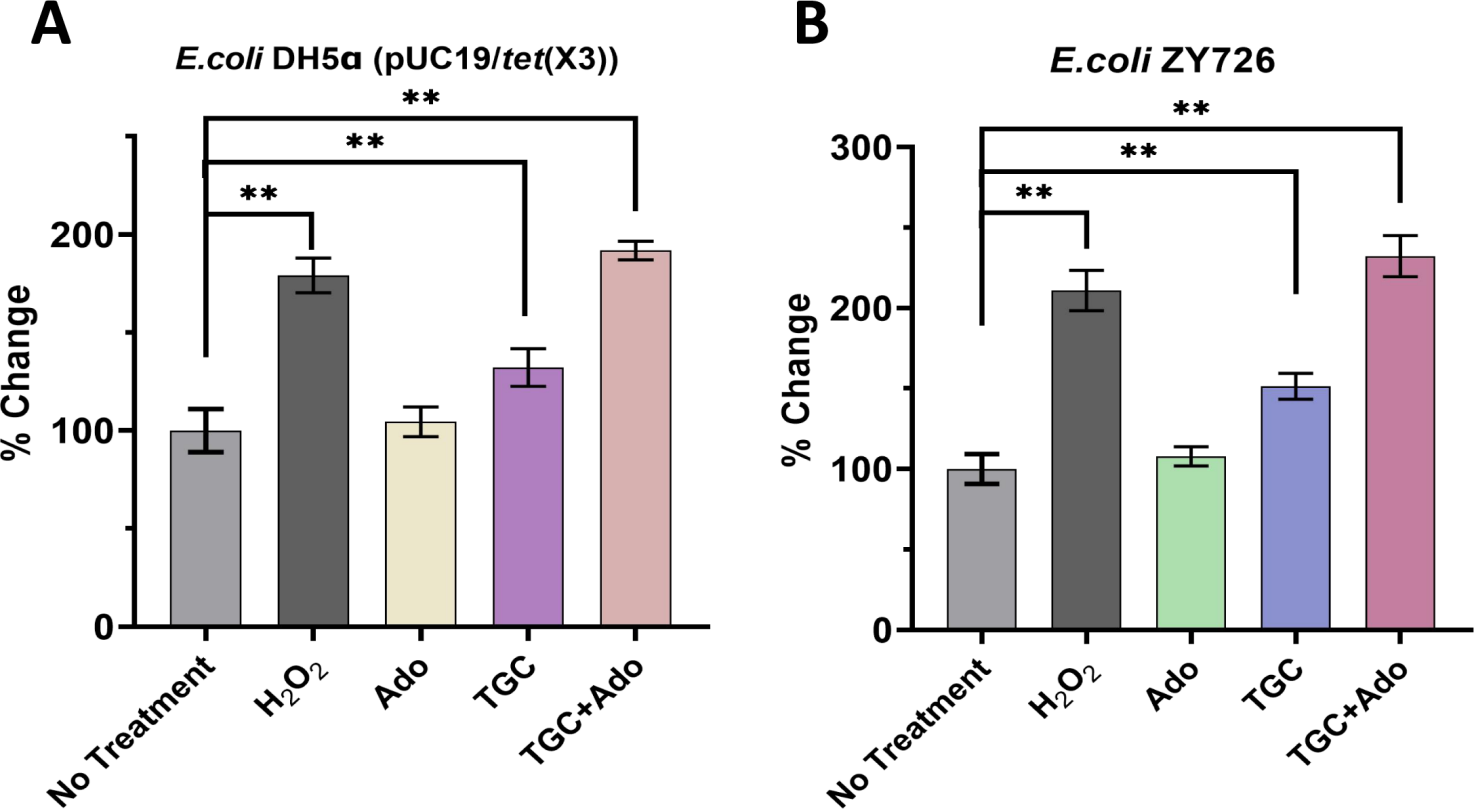
Co-administration of Ado and TGC leads to intracellular accumulation of ROS. (**A**) Alterations in intracellular ROS accumulation in engineered strains. (**B**) Modifications in intracellular ROS accumulation in natural *tet*(X3)-carrying strain. *N* = 3 independent experiments, each performed in duplicate, bars indicate means, and error bars indicate standard deviation, ** represents *P*＜0.01.

The accumulation of Reactive Oxygen Species (ROS) within bacterial cells has been established as a pivotal mechanism underlying the bactericidal action of diverse antimicrobial agents. Our previous study further confirmed that the generation of ROS is a crucial aspect of the bactericidal mechanism exhibited by β-lactam antibiotics, while also identifying the ratio between reduced glutathione and oxidized glutathione as a reliable indicator for assessing intracellular ROS production (18). Building upon these findings, we conducted further investigations to explore the potential synergistic effects of TGC and Ado on intracellular ROS formation in bacterial cells. The levels of intracellular ROS were quantified using the fluorescent probe 2´,7´-dichlorodihydrofluorescein diacetate (DCFH-DA). The results presented in Fig. 4A and B demonstrate a marked increase in intracellular ROS accumulation when TGC is combined with Ado in both the engineered strain *E. coli* DH5α (pUC19/*tet*(X3)) and *tet*(X3) gene-positive strain *E. coli* ZY726 (*P* < 0.01). In the *E. coli* DH5α (pUC19/*tet*(X3)) strain, H_2_O_2_ treatment resulted in a significant increase in ROS levels of approximately 79.18 ± 7.27% compared to the untreated control group; TGC treatment significantly boosted ROS by around 32.12 ± 7.86% compared to the untreated control group (*P* < 0.05). Strikingly, the combination of TGC and Ado induced the highest ROS accumulation, nearly doubling the levels observed in the untreated samples (91.85 ± 3.89 %) (*P*＜0.05) (Fig. 4A). A similar trend was observed in *E. coli* ZY726. H_2_O_2_ exposure led to a substantial 110.93 ± 10.20 % increase in ROS compared to the untreated control group. Ado had little impact, maintaining ROS at levels comparable to the untreated control (*P* > 0.05). TGC treatment, however, caused a robust 51.54 ± 6.54 % rise in ROS compared to the untreated control group (*P*＜0.05). Remarkably, the co-treatment of TGC and Ado resulted in the most significant effect, increasing ROS levels by approximately 132.32 ± 10.39 % compared to the untreated condition (*P*＜0.05) (Fig. 4B). These findings underscore the potent synergistic effect of combining TGC and Ado in promoting intracellular oxidative stress, a mechanism that appears to be conserved across both the natural *tet*(X3)-carrying and engineered *E. coli* strains examined.

### The effect of ROS scavengers on the reversal of tigecycline resistance in *tet*(X3)-positive *E. coli*

**Fig 5:**
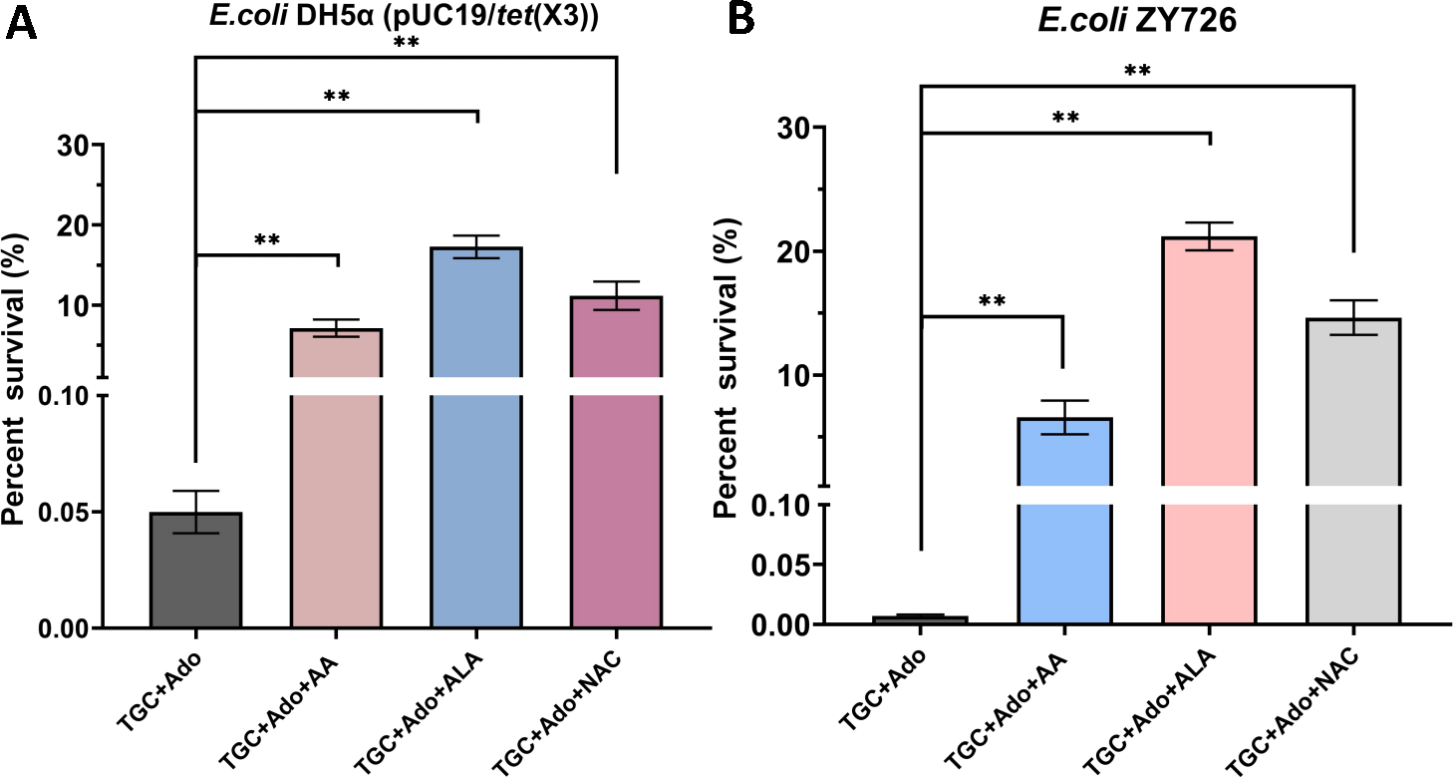
The effect of ROS scavengers on the reversal of tigecycline resistance in *tet*(X3)-positive *E. coli.* (**A**) Impact of ROS inhibitors on the survival rate of engineered strains. (**B**) Influence of ROS inhibitors on the survival rate of natural *tet*(X3)-carrying strain. AA indicates ascorbic acid, ALA indicates α-lipoic acid, and NAC indicates N-acetylcysteine. *N* = 3 independent experiments, each performed in duplicate, bars indicate means, and error bars indicate standard deviation, ** represents *P*＜0.01.

To further validate the pivotal role of increased ROS accumulation in the bactericidal action of the TGC-Ado combination, we employed three distinct ROS scavengers: ascorbic acid (AA), N-acetylcysteine (NAC), and α-lipoic acid (ALA) (Fig. 5A and B). The selection of these inhibitors was based on their unique capabilities in efficiently eliminating ROS, thereby facilitating a comprehensive evaluation of the involvement of ROS in the synergistic killing process. The experimental results presented in Fig. 5A and B clearly demonstrate that the addition of three distinct ROS inhibitors—AA, ALA, and NAC—substantially attenuated the bactericidal effect exerted by the combination of TGC and Ado in both the *E. coli* DH5α (pUC19/*tet*(X3)) and ZY726 strains (*P* < 0.01). In the DH5α (pUC19/*tet*(X3)) strain, the TGC+Ado treatment resulted in the lowest bacterial survival rate of just 0.05 ± 0.01%. However, the addition of AA, ALA, or NAC significantly improved survival, with rates reaching 7.17 ± 0.87%, 17.29 ± 1.14%, and 11.20 ± 1.45%, respectively (*P* < 0.01 for all comparisons) (Fig. 5A). A similar trend was observed in *E. coli* ZY726. The TGC+Ado combination again proved highly bactericidal, reducing survival to just 0.0069 ± 0.0010%. Yet, the presence of the ROS inhibitors markedly enhanced bacterial persistence, with survival rates increasing to 6.55 ± 1.12% (AA), 21.18 ± 0.91% (ALA), and 14.63 ± 1.14% (NAC) (*P* < 0.01 for all comparisons) (Fig. 5B). Notably, the ALA-supplemented treatment exhibited the most pronounced protective effect in both *E. coli* strains, consistently yielding the highest survival rates among the ROS inhibitor conditions tested. These results underscore the critical role of ROS in mediating the bactericidal synergy between TGC and Ado. The significant attenuation of this effect by AA, ALA, and NAC suggests that these compounds may act to mitigate the accumulation of ROS, thereby safeguarding the bacteria against the detrimental oxidative stress induced by the TGC+Ado combination.

### Intracellular oxidative stress damage in *tet*(X3)-positive *E. coli*

**Fig 6:**
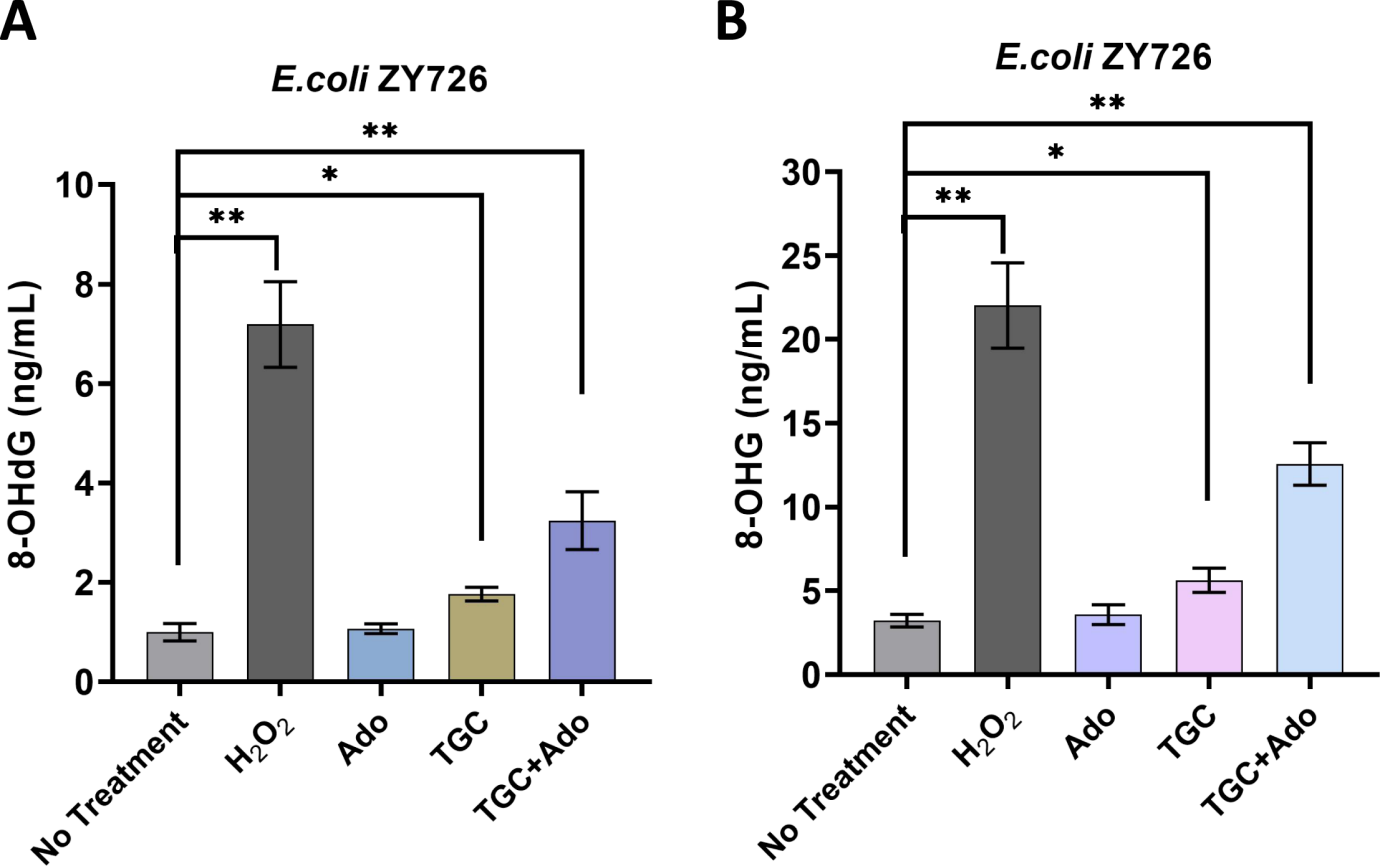
Intracellular oxidative stress damage in *tet*(X3)-positive *E. coli.* (**A**) Effects of adenosine and tigecycline combination on intracellular 8-OHdG levels. (**B**) Effects of adenosine and tigecycline combination on intracellular 8-OHG levels. *N* = 3 independent experiments, each performed in duplicate, bars indicate means, and error bars indicate standard deviation, * represents *P*＜0.05, ** represents *P*＜0.01.

The presence of 8-hydroxydeoxyguanosine (8-OHdG) and 8-hydroxyguanosine (8-OHG) in DNA or RNA is widely utilized as a biomarker for oxidative stress, which can result from oxidation at the nucleic acid level (19). To quantify the levels of 8-OHdG and 8-OHG, two distinct enzyme-linked immunosorbent assay (ELISA) assays targeting these modifications were employed. While the Ado-treated did not exhibit a significant alteration in 8-OHdG content compared to the untreated group, the TGC-treated displayed a noteworthy increase in 8-OHdG content (*P* < 0.05). These findings suggest that TGC alone can induce oxidative stress, leading to DNA damage, which is consistent with previous reports indicating that bactericidal antibiotics can elicit oxidative damage in bacteria (20). The combination of TGC and Ado resulted in a significant increase in the content of 8-OHdG (*P* < 0.01) (Fig. 6A), indicating enhanced oxidative damage. The aforementioned observation implies a synergistic effect, whereby Ado modulates cellular metabolism, potentially resulting in ATP depletion and alteration of redox states. Similarly, the combined use of TGC and Ado exhibited a significantly elevated 8-OHG content (*P* < 0.01) (Fig. 6B), implying that metabolic changes may further exacerbate oxidative stress and nucleic acid damage. These findings suggest that the combination of antibiotics and metabolic perturbations can exacerbate oxidative damage to nucleic acids, thereby contributing to the underlying mechanisms of antibiotic-induced cell death.

### Co-administration of Ado and TGC induces morphological alterations in bacteria harboring the *tet*(X3) gene

**Fig 7:**
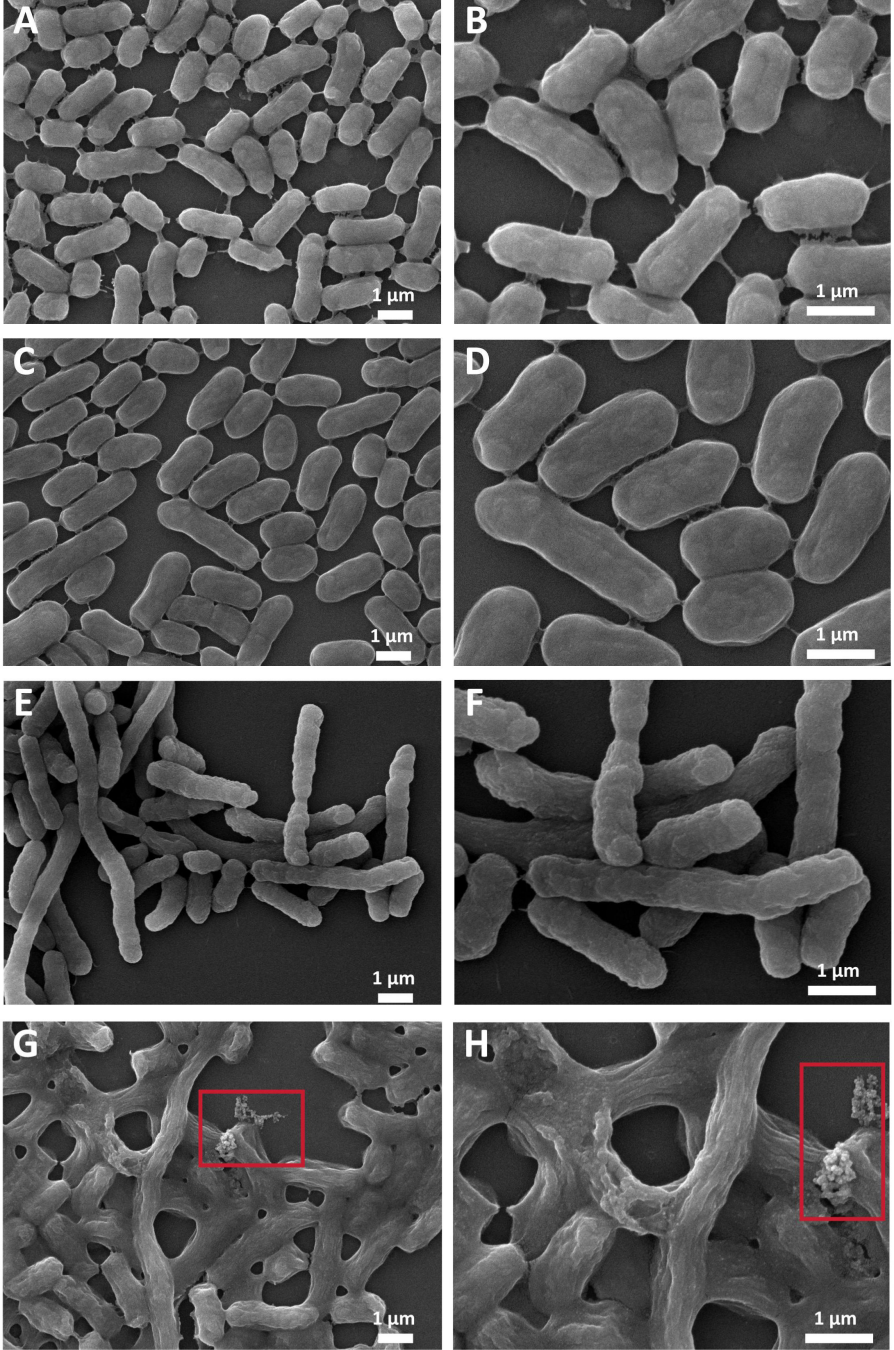
Scanning electron microscopic characterization of morphological changes in *tet*(X3)-harboring *E. coli* DH5α (pUC19/tet(X3)). (**A**) Morphological changes in the control group bacteria (10,000×). (**B**) Morphological changes in the control group bacteria (20,000×). (**C**) Morphological changes in the adenosine-treated group bacteria (10,000×). (**D**) Morphological changes in the adenosine-treated group bacteria (20,000×). (**E**) Morphological changes in the TGC-treated group bacteria (10,000×). (**F**) Morphological changes in the TGC-treated group bacteria (20,000×). (**G**) Bacterial morphological changes in the adenosine and TGC combination treatment group (10,000×). (**H**) Bacterial morphological changes in the adenosine and TGC combination treatment group (20,000×). The red boxes delineate areas demonstrating bacterial cytoplasmic content leakage.

Scanning electron microscopy (SEM) was utilized to examine bacterial morphological alterations under various treatment conditions in order to explore the effects of combining TGC and Ado on *tet*(X3)-positive *E. coli*. The majority of bacteria retained their typical rod-shaped morphology with intact cell walls and smooth surfaces in both the untreated control group and samples treated individually with Ado (Fig. 7A through D). The surface of *E. coli* cells displays wrinkling and shrinkage upon treatment with TGC, resulting in a more rugged and irregular appearance compared to untreated cells (Fig. 7E and F). However, despite these observed alterations on the cell surface, the fundamental rod-shaped morphology of individual bacterial cells remains intact. However, the combination of TGC and Ado resulted in notable morphological alterations. A majority of bacteria exhibited significant contraction of their cell walls, leading to a reduction in cell volume and an irregular surface. In certain extreme cases, complete bacterial lysis and leakage of intracellular contents were even observed (Fig. 7G and H).

### *In vivo* therapeutic efficacy of Ado demonstrates remarkable synergistic effects

**Fig 8:**
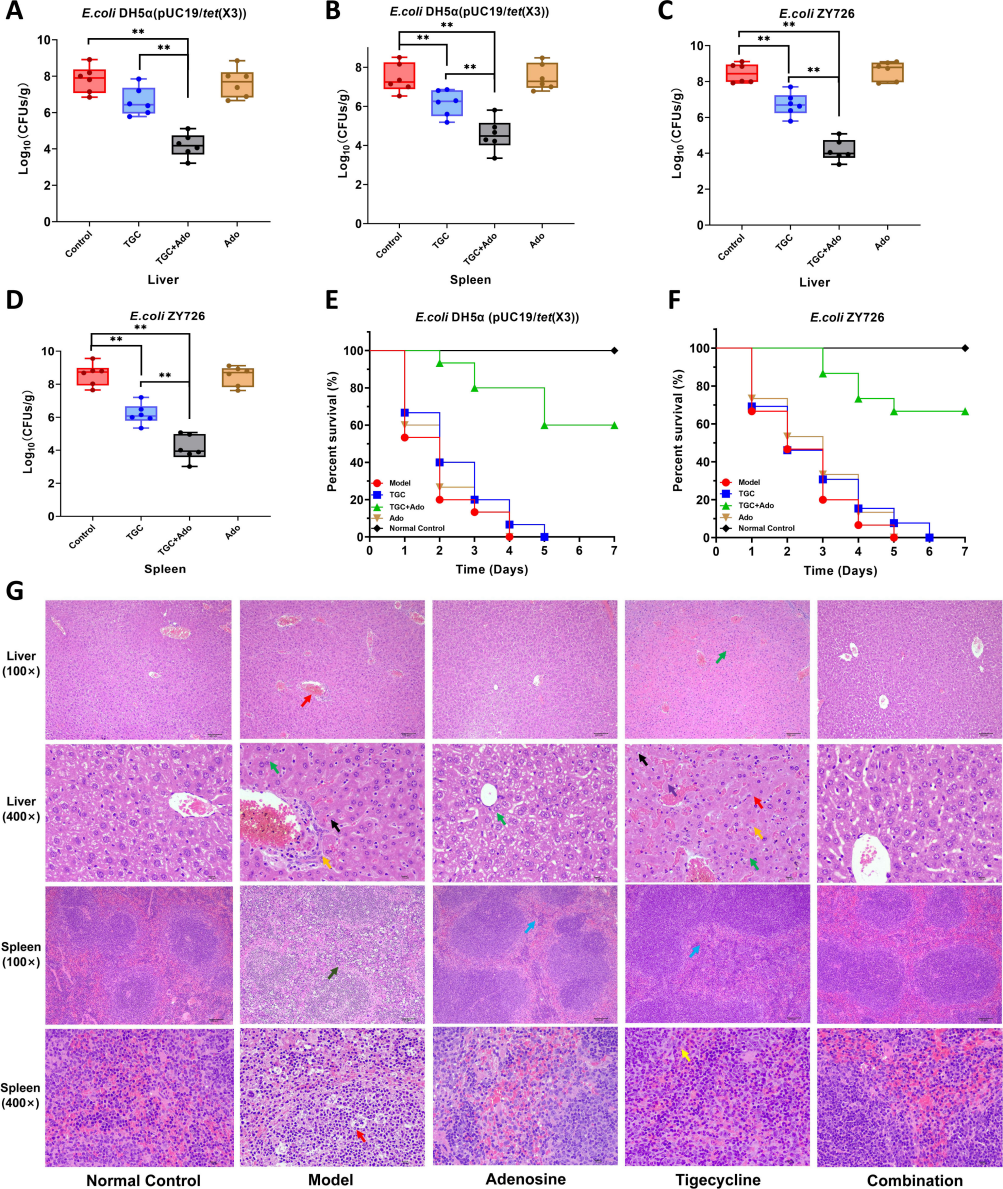
*In vivo* therapeutic efficacy of Ado demonstrates remarkable synergistic effects. (**A**) The bacterial load of engineered strain in the liver of mice (*N* = 6). (**B**) The bacterial load of engineered strain in the spleen of mice (*N* = 6). (**C**) The bacterial load of natural *tet*(X3)-carrying strain in the liver of mice (*N* = 6). (**D**) The bacterial load of natural *tet*(X3)-carrying strain in the spleen of mice (*N* = 6). (**E**) Seven-day survival rate of mice infected with engineered strains (*N* = 15). (**F**) Seven-day survival rate of mice infected with natural *tet*(X3)-carrying strain (*N* = 6). (**G**) Histopathological observation of mouse liver and spleen. ** represents *P*＜0.01. Bars indicate means, and error bars indicate standard deviation. The “normal control” group consisted of mice injected with physiological saline, while the “model” group comprised mice with induced bacterial resistance that received no therapeutic intervention. The red arrows indicate areas of necrosis, green arrows denote lipid degeneration, blue arrows highlight extramedullary hematopoiesis in the spleen, orange arrows indicate fibroblasts, yellow arrows represent neutrophil proliferation in the splenic red pulp, purple arrows indicate areas of congestion, black arrows point to lymphocytes, and gray arrows indicate loss of splenic marginal zone.

We investigated the antimicrobial mechanisms of Ado in combination with TGC, revealing a significant synergistic antibacterial effect *in vitro* and demonstrating effective bactericidal action *in vitro* (Fig. 3A through H). These findings suggest that Ado can reverse TGC resistance mediated by the *tet*(X3) gene *in vitro*. However, it remains unclear whether the resistance-reversing effect of Ado against *tet*(X3)-carrying *E. coli* is similarly effective *in vivo*. To address this crucial question, we designed an intraperitoneal infection model using *E. coli* DH5α (pUC19/*tet*(X3)) and *E. coli* ZY726 to evaluate the efficacy of different treatment regimens *in vivo*. The results indicated that, in the case of *E. coli* DH5α (pUC19/*tet*(X3)) infection, the TGC group exhibited a modest reduction in bacterial counts in the liver; however, these counts remained relatively elevated, suggesting that TGC had a discernible inhibitory effect on *E. coli* but not a statistically significant one. The Ado group demonstrated comparable liver bacterial counts to those of the control group, implying that Ado alone did not exert a substantial impact on *E. coli* growth. In contrast, combined treatment with TGC and Ado resulted in significantly lower bacterial counts in the liver than other groups did, implying that synergy between TGC and Ado markedly enhanced their inhibitory effect on *E. coli* growth (Fig. 8A). Regarding bacterial counts in the spleen, although reduced compared to those of the control group, TGC values remained elevated at approximately 6–7 log colony-forming unit (CFU)/g. In contrast, Ado’s bacterial count was comparable to that of controls, indicating no significant impact on *E. coli* growth. However, noteworthy synergistic effects were observed upon co-administration of TGC and Ado, resulting in significantly lower bacterial loads (4–5 log CFU/g) (*P* < 0.01) (Fig. 8B). A similar trend was observed in the infection model using natural *tet*(X3)-carrying strain, where co-administration of TGC and Ado significantly diminished bacterial loads in the liver and spleen of mice post-treatment (*P* < 0.01). (Fig. 8C and D).

We also prepared tissue sections from the liver and spleen of mice infected with natural *tet*(X3)-carrying strain for pathological examination (Fig. 8G). Histopathological analysis revealed that the model group exhibited hepatic steatosis, hepatocyte necrosis, inflammatory cell infiltration, and hepatic fibrosis, while the spleen showed lymphocyte necrosis and loss of the marginal zone. In the Ado treatment group, hepatocytes displayed vacuolar degeneration, and there was an increase in extramedullary hematopoiesis in the spleen. The TGC treatment group demonstrated hepatic steatosis, hepatocyte necrosis, inflammatory cell infiltration, fibrosis, and congestion of hepatic sinusoids in the liver, along with increased extramedullary hematopoiesis in the spleen. In contrast, tissue sections from the combination treatment group closely resembled those of normal mice.

In the 7-day survival rate experiment, all mice in the untreated control group and the Ado treatment group succumbed to infection within 4 days post-infection when using *E. coli* DH5α (pUC19/*tet*(X3)) for the intraperitoneal infection model (Fig. 8E). This outcome suggests that these two interventions were ineffective in inhibiting bacterial growth or mitigating the lethal effects of the infection. Similarly, TGC treatment exhibited limited efficacy in this type of infection, as evidenced by a relatively short survival time with all mice perishing within 5 days, likely due to bacterial resistance mechanisms. In contrast, the combination treatment group demonstrated superior performance compared to other groups. By day 5 of infection, the survival rate among mice receiving combined therapy reached 60.0%, and no further mortalities occurred thereafter, indicating not only an improvement in short-term survival but also enhanced long-term survivability (Fig. 8E). In the intraperitoneal infection model using *E. coli* ZY726, all mice in the untreated control group and Ado treatment group succumbed within 5 days, while all mice in the TGC group perished within 6 days (Fig. 8F). In contrast, the combination treatment exhibited a survival rate of 66.67% within 5 days, with no additional fatalities observed thereafter. These findings further substantiate the efficacy of combination therapy, underscoring its significant antibacterial activity and protective effects against this type of intraperitoneal infection.

## DISCUSSION

The *tet*(X3) gene encodes a flavin-dependent monooxygenase that catalyzes the hydroxylation of tetracycline antibiotics, rendering them inactive. The gene is often located on plasmids and can be transferred horizontally via mobile genetic elements, facilitating its spread among pathogens. This mechanism poses a significant threat to the clinical efficacy of tigecycline, a last-line antibiotic for multidrug-resistant infections. The metabolic change of *tet*(X3)-harboring *E. coli* reveals intricate remodeling of cellular pathways, underscoring the complex interplay between antibiotic resistance and bacterial metabolism. The upregulation of key metabolites, such as pantothenate, tartaric acid, and folic acid, suggests an increased demand for cofactors and energy sources to support the enhanced enzymatic activities conferred by the *tet*(X3) gene (21). Conversely, the downregulation of nucleotide and amino acid-associated metabolites may signify a metabolic trade-off or resource reallocation strategy aimed at sustaining the antibiotic resistance phenotype (22). These substantial alterations in the metabolic landscape indicate a profound remodeling of metabolic pathways, highlighting the intimate connection between antibiotic resistance and cellular metabolism (14). Clustering analysis further substantiates the profound metabolomic differences between *tet*(X3)-positive *E. coli* and their susceptible counterparts. Among the upregulated metabolites, the increase in pantothenate, a precursor of coenzyme A, is associated with enhanced bacterial energy metabolism. The concurrent upregulation of ADP and ADP-glucose, coupled with elevated pantothenate levels, suggests that bacteria may have augmented their energy production and storage capabilities. The increase in citrate levels also implies the potential activation of the TCA cycle, further supporting the observed energy metabolism remodeling (23). This metabolic shift represents an adaptive strategy employed by bacteria to cope with the heightened energy demands associated with antibiotic resistance mechanisms. Furthermore, the upregulation of folic acid and folinic acid, alongside the downregulation of nucleotide precursors, indicates substantial alterations in bacterial nucleic acid metabolism. These changes may be linked to enhanced DNA repair capacity, enabling bacteria to better withstand antibiotic-induced genomic insults (24). Among the downregulated metabolites, the decrease in several amino acids may reflect changes in bacterial protein synthesis patterns. This adaptation could serve as a strategic response to conserve energy or modulate the protein composition for coping with antibiotic-induced stress (25). Alterations in specific metabolites, such as gallic acid, may be associated with the regulation of bacterial redox balance. The regulatory process could aid bacteria in managing the oxidative stress induced by antibiotics. Maintaining redox homeostasis is crucial for bacterial survival and function under antibiotic-induced stress. Additionally, changes in metabolites like deoxycholic acid may indicate modifications in bacterial membrane lipid composition, potentially impacting the permeability of cell membranes and influencing the entry and efflux of antibiotics (26). Bacteria frequently modulate membrane properties to reduce intracellular antibiotic accumulation and enhance resistance. The downregulation of nucleotide precursors, including adenine, adenosine, and cytidine, suggests alterations in bacterial nucleic acid metabolism. Additionally, a decrease in fumarate levels may indicate the inhibition of specific steps within the tricarboxylic acid cycle, further influencing nucleotide metabolism.

The *tet*(X3) gene triggers extensive metabolic remodeling in bacteria, leading to a sophisticated network of adaptations that bolster antibiotic resistance. This intricate process encompasses strategic modifications to key metabolic pathways, optimizing energy utilization, membrane permeability, DNA repair, protein synthesis, and oxidative stress management. The upregulation of pantothenate, a CoA precursor, directly impacts a myriad of essential metabolic reactions, empowering bacterial growth and survival under antibiotic pressure (27). The observed disturbances in purine metabolism suggest adaptations in nucleic acid composition and DNA maintenance mechanisms. Modifications to the TCA cycle further indicate a strategic recalibration of energy metabolism, enabling bacteria to optimize energy utilization in the face of antibiotic stress (28). Perturbations in amino acid metabolism pathways, including arginine, histidine, alanine, aspartate, and glutamate, have the potential to influence bacterial protein synthesis, modification, and membrane properties—all of which can contribute to the development of antibiotic resistance (29). Metabolic perturbations in vitamin B6, folate, and nicotinate pathways suggest a comprehensive restructuring of bacterial metabolic networks in response to combinatorial stress. These alterations potentially modulate cofactor availability and oxidative defense mechanisms, impacting critical cellular processes including cell wall synthesis and nucleic acid metabolism.

In the checkerboard assay, all tested combinations demonstrated FICI values below 0.5, providing robust evidence for a significant synergistic interaction between Ado and both TET antibiotics. This synergy implies the possibility of achieving therapeutic effectiveness at considerably reduced antibiotic dosages to mitigate potential toxicity risks and minimize resistance development. The observed synergy between Ado and both TGC and TET against *tet*(X3)-positive strains as well as *E. coli* ZY726 underscores its broad-spectrum adjunctive capacity in augmenting the efficacy of TET antibiotics. The dose-dependent characteristic of this synergistic effect indicates that optimizing Ado dosage holds promise for maximizing therapeutic benefits in clinical settings. Ado significantly augments the bactericidal activity of TGC and TET in combating *tet*(X3)-harboring *E. coli*. The co-administration of Ado leads to a significantly lower survival rate compared to the use of antibiotics alone across all experimental groups. The synergistic effect of Ado was observed early on (2–4 h) and became more pronounced over time. Interestingly, this synergy was evident in both engineered strain and natural *tet*(X3)-carrying strain, although it was generally stronger in natural *tet*(X3)-carrying strain due to their long-term natural selection and evolution, resulting in complex interactions with their environment and other organisms (30). Natural *tet*(X3)-carrying strains have optimized and retained genes and metabolic pathways favorable for synergy during this process, making them more competitive within communities (31). In contrast, engineered strains often focus only on specific target genes or metabolic pathways while neglecting the overall complexity of gene networks involved in synergy, which may lead to poor performance or unexpected negative effects (32).

The combination of Ado and TGC has been found to potentiate the antibacterial effect, resulting in a substantial elevation of ROS levels. Notably, Ado alone does not directly induce ROS generation, indicating its inability to independently trigger oxidative stress. These findings imply that Ado may synergistically enhance the cellular oxidative stress response under the influence of TGC. While TGC alone also increases ROS production, the effect is considerably less pronounced than with the combination therapy. Previous studies have demonstrated that the activation of signaling pathways, such as AMPK (AMP-activated protein kinase), is intricately associated with cellular energy metabolism and may augment the cellular response to oxidative stress (33). The observed significant elevation in intracellular ROS levels upon combining Ado and TGC is potentially attributed to Ado-induced activation of AMPK and other signaling pathways, thereby further amplifying ROS generation under the influence of TGC (34). This finding aligns with previous research indicating that AMPK activation can stimulate mitochondrial biogenesis and function, consequently leading to increased ROS production (35). Ado may also induce ROS generation by modulating cell membrane permeability or intracellular calcium ion concentrations. Alterations in cell membrane permeability can impact the entire cellular environment, thereby modifying the oxidative stress response (36), and changes in calcium ion concentration are also linked to various signaling pathways associated with oxidative stress (37). The substantial decrease in the bactericidal effect observed with all three distinct mechanisms of ROS inhibitors (ascorbic acid, N-acetylcysteine, α-lipoic acid) suggests that the primary mechanism underlying the killing action of the TGC-Ado combination is the accumulation of ROS. The differential inhibitory effects suggest that the combination of TGC and Ado may enhance ROS generation or weaken bacterial antioxidant defenses through multiple pathways. This aligns with Dwyer et al., who found that certain antibiotics can simultaneously modulate various cellular processes, resulting in excessive ROS accumulation (38).

We demonstrate that the combination of TGC and Ado can synergistically enhance oxidative damage to DNA and RNA, as evidenced by the increased levels of 8-OHdG and 8-OHG, respectively. This observation is consistent with previous reports indicating that bactericidal antibiotics, such as TET, can induce oxidative stress and subsequent nucleic acid damage in bacteria (39). The findings suggest that the modulation of cellular metabolism by Ado may contribute to this synergistic effect. Ado has been shown to alter cellular energy homeostasis, leading to ATP depletion and changes in redox states (40). The metabolic perturbations could exacerbate the oxidative stress induced by TET, resulting in enhanced DNA and RNA damage. The increased levels of 8-OHdG and 8-OHG observed in the present study serve as reliable biomarkers of oxidative stress and nucleic acid damage (41). These oxidative modifications have the potential to hinder diverse cellular processes such as DNA replication, transcription, and translation. Ultimately, they play a significant role in the underlying mechanisms of antibiotic-induced cell death (42).

Bacterial cell wall contraction and increased membrane permeability likely represent cellular stress responses to combinatorial drug pressure. The observed membrane alterations are consistent with ROS-mediated lipid damage, as previously documented by Fang et al., who demonstrated that direct oxidative assault compromises membrane integrity (43). The lysis of bacteria and release of intracellular contents serve as direct evidence for the bactericidal effect. This observation aligns with the seminal work by Imlay and Linn, who initially proposed the concept of “oxidative killing,” wherein excessive ROS can inflict irreversible damage on bacterial DNA, proteins, and lipids, ultimately culminating in cell death (44). The observed morphological changes are highly consistent with the previously reported synergistic antibacterial effect and the enhanced accumulation of ROS. We propose that TGC, as a protein synthesis inhibitor, may disrupt the expression of proteins involved in cell wall synthesis, thereby compromising the integrity of the cell wall structure. Meanwhile, Ado may exacerbate the cellular stress response by modulating the bacteria’s energy metabolism or signaling pathways, thereby further compromising their ability to maintain normal cell morphology and membrane integrity. The observed shrinkage, indentation, and rupture of the bacterial cell membrane in our SEM analysis are particularly noteworthy due to their strong consistency with previous findings emphasizing the pivotal role of ROS accumulation in the bactericidal process facilitated by the TGC-Ado combination. This phenomenon is likely attributed to ROS attacking the bacterial cell membrane, as supported by Van Acker et al.’s research demonstrating that ROS can induce lipid peroxidation and disrupt membrane structure (45). Additionally, our observations align with the findings of Belenky et al., who utilized atomic force microscopy to observe notable morphological alterations and membrane impairment in bacteria subjected to antibiotic-induced oxidative stress (25). The aforementioned finding further supports our hypothesis that the synergistic bactericidal effect of TGC and Ado may be attributed to the enhancement of ROS production.

The *in vitro* results revealed a significant synergistic antibacterial effect between Ado and TGC or TET, suggesting that Ado can effectively reverse TGC resistance mediated by the *tet*(X3) gene (46). Importantly, the *in vivo* findings from the intraperitoneal infection model provide compelling evidence that this synergistic combination therapy exhibits superior efficacy compared to either treatment alone, successfully reducing bacterial loads in the liver and spleen and enhancing the survival rate of infected mice. The findings highlight the clinical potential of this approach, particularly in treating infections caused by *tet*(X3)-carrying pathogens, which are increasingly prevalent in healthcare settings. Future clinical studies could explore dosing regimens and safety profiles to facilitate the translation of this therapeutic strategy into clinical practice. The mechanism underlying the resistance-reversing effect of Ado is not fully elucidated, but it is postulated to involve modulation of cellular stress responses and energy metabolism pathways (47). Ado has been shown to inhibit the expression of efflux pumps and other resistance determinants, thereby potentiating the activity of antibiotics (48). Furthermore, the ability of Ado to enhance mitochondrial function and ATP production may sensitize bacteria to the bactericidal effects of TGC, which targets protein synthesis (49). This mechanistic insight provides a foundation for developing adjunctive therapies that target bacterial metabolism to overcome resistance, which could be particularly valuable in clinical settings where conventional antibiotics alone are ineffective. The superior *in vivo* efficacy of the Ado-TGC combination is particularly noteworthy, as it effectively overcame the resistance conferred by the *tet*(X3) gene and significantly reduced bacterial burdens in the liver and spleen of infected mice. This suggests that the Ado-TGC combination could be a promising candidate for clinical trials, especially in treating severe infections caused by multidrug-resistant gram-negative bacteria. The histopathological analysis further underscores the protective effects of the combination treatment, as it mitigated the tissue damage and inflammation observed in the untreated and single-treatment groups. These results not only demonstrate the therapeutic potential of this approach but also emphasize its potential to reduce morbidity and mortality in patients with resistant infections. Future studies should investigate the feasibility of incorporating this combination into existing antibiotic treatment protocols in clinical practice.

**Fig 9:**
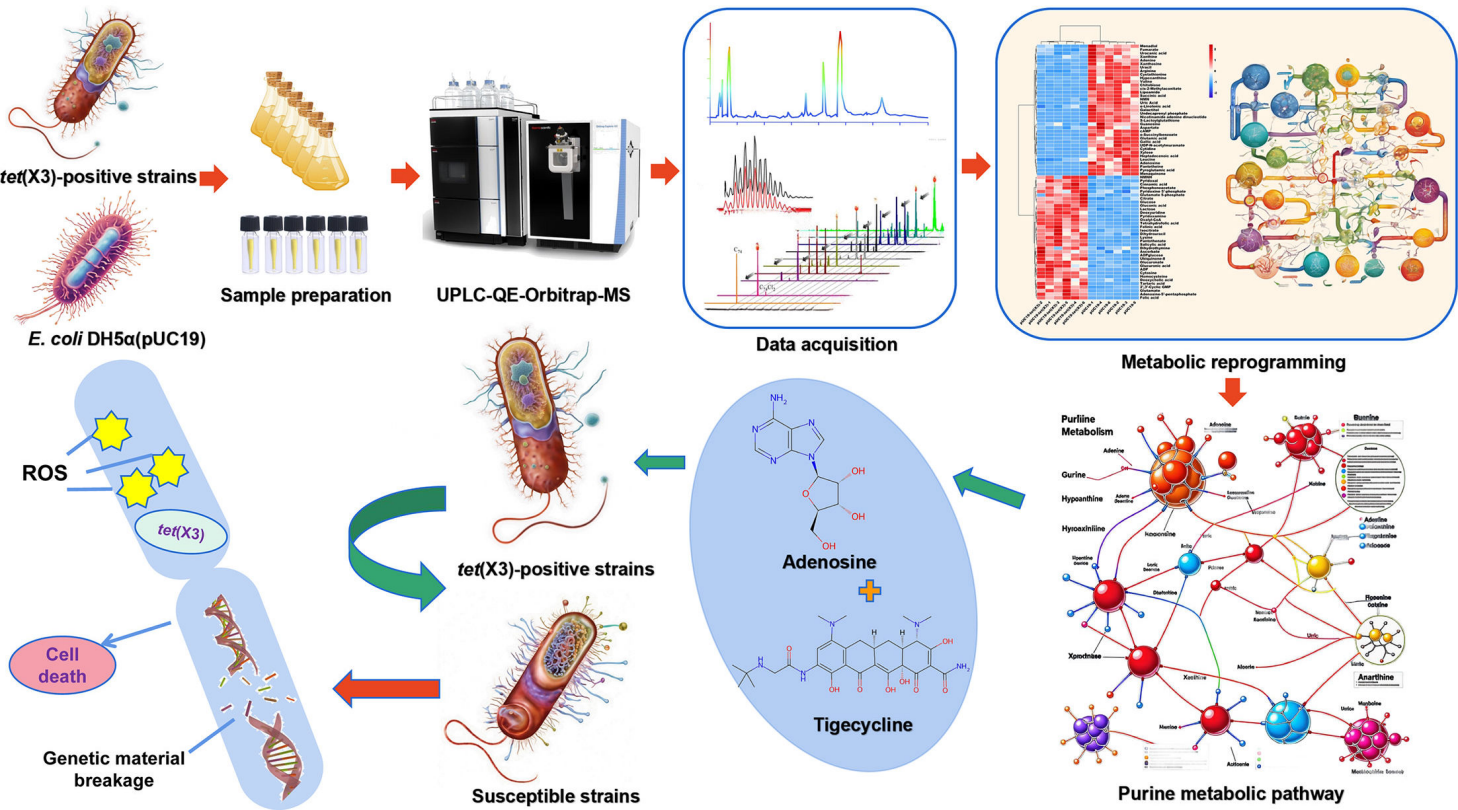
Metabolic reprogramming strategy for metabolite screening in tigecycline-resistant *tet*(X3)-positive bacteria and elucidation of their synergistic bactericidal mechanism. The process begins with the cultivation of strains, followed by sample preparation for metabolomic analysis. The prepared samples are analyzed using UPLC-QE-Orbitrap-MS to profile metabolites. Visual data comparisons reveal significant metabolic reprogramming in *tet*(X3)-positive strains, particularly in response to adenosine and tigecycline, suggesting potential antibiotic resistance mechanisms. Pathway mapping indicates alterations in the purine metabolic pathway, illustrating the adaptive responses of these strains under antibiotic stress.

In conclusion, this study investigates a novel reversible metabolite, Ado, associated with *tet*(X3)-mediated TGC resistance and demonstrates its ability to reverse antibiotic resistance by modulating the cellular redox state. We observed that the combination of Ado and TGC significantly elevates intracellular ROS levels, leading to DNA/RNA damage and restoring susceptibility of *tet*(X3)-positive bacteria to TGC. The synergistic effect enhances the bactericidal activity of the antibiotic. Furthermore, the murine infection model validates the potential therapeutic application of combining Ado and TGC (Fig. 9). Overall, our study proposes an innovative strategy utilizing exogenous metabolites to overcome *tet*(X3)-mediated TGC resistance, providing a promising approach for addressing clinical infections caused by *tet*(X3)-positive bacteria.

## MATERIALS AND METHODS

### Bacterial strains and reagents

The bacterial strains utilized in this study comprised *E. coli* DH5α (pUC19), *E. coli* DH5α (pUC19/*tet*(X3)), and natural *tet*(X3)-carrying strain *E. coli* ZY726 (*tet*(X3) gene-positive *E. coli* isolate originally recovered from cattle feces collected at a dairy farm in Gansu Province, China). Unless otherwise noted, all bacterial strains were cultivated under the conditions of Luria-Bertani (LB)/Mueller-Hinton medium at a temperature of 37℃. Exogenous metabolites, antibiotics, and ROS inhibitors were purchased from Solarbio (Beijing).

### Construction of engineered bacteria harboring the *tet*(X3) gene

Using the natural *tet*(X3)-carrying *Acinetobacter baumannii* isolated from clinical samples in our laboratory as a template, we performed restriction digestion and ligation of the target gene with the pUC19 plasmid to construct a cloning vector. Subsequently, this vector was introduced into *E. coli* DH5α, resulting in the generation of an engineered strain named *E. coli* DH5α (pUC19/*tet*(X3)). Additionally, we also transformed *E. coli* DH5α with the empty pUC19 plasmid lacking the target gene, generating *E. coli* DH5α (pUC19). Detailed results can be found in the Supplementary Materials (Fig. S2A and B).

### Metabolic profiling and metabolomics analysis

The bacterial strains *E. coli* DH5α (pUC19/*tet*(X3)) and *E. coli* DH5α (pUC19) were quenched using a methanol/ethylene glycol mixture (−60°C) and subsequently washed with 0.85% NaCl solution. The resulting cell pellets were extracted with boiling ethanol/water (75:25, vol/vol, 95°C). Metabolite separation was performed using both reversed-phase (RP) and hydrophilic interaction liquid chromatography (HILIC) techniques, followed by detection with a quadrupole-Orbitrap mass spectrometer in positive and negative electrospray ionization modes (Thermo Scientific). For RP separation, a BEH Shield RP C18 column (2.1 × 100 mm, 1.7 µm) (Waters) was utilized with mobile phases comprising A (0.1% formic acid in water) and B (0.1% formic acid in acetonitrile) for ESI+ mode, and A (5 mM ammonium acetate in water) and B (5 mM ammonium acetate in acetonitrile) for ESI− mode. The gradient profile was as follows: 0–1 min, 98% A; 1–10 min, 98%–60% A; 10–11 min, 60%–2% A; 11–13 min, 2% A; 13–13.1 min, 2%–98% A; 13.1–15 min, 98% A. For HILIC separation, a BEH Amide column (100 × 2.1 mm, 1.7 µm) (Waters) was employed, using solvents A (0.1% formic acid + 5 mM ammonium acetate in water) and B (0.1% formic acid in acetonitrile) for ESI + mode, and A (5 mM ammonium acetate in water) and B (5 mM ammonium acetate in acetonitrile) for ESI− mode. The gradient was set as follows: 0–2 min, 5% A; 2–8 min, 5%–30% A; 8–9 min, 30%–50% A; 9–11 min, 50% A; 11–12 min, 50%–5% A; 12–15 min, 5% A. The flow rate was maintained at 0.3 mL/min with an injection volume of 2 µL. Orbitrap mass spectrometry parameters included a capillary temperature of 300°C, spray voltage of 2.8 kV, resolution set to 70,000, and a scan range between 150 and 2,000 m/z, with stepped collision energy at 20, 40, and 60 eV. Data processing was conducted using Progenesis QI software, applying statistical criteria (*P* < 0.05, coefficient of variation (CV) <30%, variable importance in projection (VIP) >1, and fold change ≥1.5) to identify differentially abundant metabolites. Metabolite identification was achieved by database searches against the ECMDB (http://www.ecmdb.ca).

### Synergistic bacteriostasis experiment

The checkerboard microdilution method was employed to assess the MICs of Ado, TGC, and TET against a range of bacterial strains. This was conducted using sterile 96-well microtiter plates filled with Mueller-Hinton broth, allowing for serial twofold dilutions of Ado and the antibiotics. Following the addition of 100 µL of a bacterial suspension at a concentration of 1 × 10^6^ CFU/mL to each well, the plates were incubated at 37°C for a duration of 16 to 24 h. The MICs were determined as the lowest concentrations at which no visible bacterial growth was observed. To evaluate potential synergistic interactions between Ado and the antibiotics against drug-resistant strains, an 8 × 10 checkerboard microdilution matrix was utilized. This approach involved mixing the compounds in twofold serial dilutions along with a bacterial suspension at 5 × 10^5^ CFU/mL. The FICI was calculated using the formula FICI = (MIC compounds in combination/MIC compound alone) + (MIC antibiotics in combination/MIC antibiotics alone). Synergism was defined as an FICI ≤0.5.

### Time-killing curve

The bacterial suspensions (1 × 10^6^ CFU/mL) were prepared in M9 minimal medium supplemented with 10 mM ammonium acetate, 1 mM MgSO_4_, and 100 µM CaCl_2_ to investigate the effects of Ado and TGC/TET on bacterial survival. Then, the suspensions were treated with either Ado, TGC/TET, or a combination of Ado and TGC/TET. TGC concentrations of 8 µg/mL for *E. coli* DH5α (pUC19/*tet*(X3)) and 32 µg/mL for *E. coli* ZY726 were used, while TET concentrations of 32 µg/mL for *E. coli* DH5α (pUC19/*tet*(X3)) and 128 µg/mL for *E. coli* ZY726 were employed. The Ado concentration was 8 mM for all treatment groups. At time points of 0, 2, 4, 6, and 8 h after treatment, culture supernatants were collected and subjected to serial dilution before being plated onto LB agar plates. The resulting bacterial colonies were counted to determine the percentage of survival for each treatment group.

### Determination of intracellular ROS accumulation

Individual colonies of *E. coli* DH5α (pUC19/*tet*(X3)) and *E. coli* ZY726 were meticulously isolated and inoculated into sterile LB broth for overnight incubation at 37℃. Subsequently, the overnight cultures were transferred to conical flasks containing LB broth and cultivated until reaching an optical density (OD_600_) of approximately 0.35. The cultures were then centrifuged at 8,000 rpm for 5 min, followed by two washes with 30 mL of sterile saline solution. The resulting pellet was resuspended in M9 minimal medium supplemented with ammonium acetate (10 mM), MgSO_4_ (1 mM), and CaCl_2_ (100 µM). TGC was added to the resuspended cultures at concentrations of 8 µg/mL for *E. coli* DH5α (pUC19/*tet*(X3)) and 32 µg/mL for *E. coli* ZY726, while Ado was included at a concentration of 8 mM. The combination of Ado and TGC is used as the synergistic experimental group, while 10 mM H_2_O_2_ serves as the positive control group. Each experimental group consisted of three replicates and underwent incubation at a temperature of 37℃ for 6 h before assessing intracellular ROS levels using the GENMED High-Quality Fluorescent Assay Kit for ROS (Genmed Scientific, China).

### Inhibition of intracellular ROS

The *E. coli* DH5α (pUC19/*tet*(X3)) and *E. coli* ZY726 were adjusted to achieve a turbidity equivalent to a McFarland standard of 0.5, and then diluted in 10 mL of M9 media at a ratio of 1:100 to approximate a concentration of 10^6^ CFU/mL. TGC was added to the media at concentrations of 8 µg/mL for *E. coli* DH5α (pUC19/*tet*(X3)) and 32 µg/mL for *E. coli* ZY726, followed by the addition of Ado at a concentration of 8 mM. Additionally, ROS inhibitors, including ascorbic acid, N-acetylcysteine, and α-lipoic acid, were incorporated into the medium at a concentration of 10 mM. Bacterial survival rates were assessed after 6 h.

### Determination of 8-OHdG and 8-OHG

Overnight cultures were diluted 1:250 in 25 mL LB medium in 250 mL baffled flasks and grown to an OD_600_ of 0.35. The cells were then treated with the respective drugs for each treatment group for 6 h, at which point they were collected and centrifuged at 4,000 rpm for 10 min in a benchtop swinging-bucket centrifuge. Following washing with phosphate buffered saline (PBS), the pellets were resuspended in 400 µL of 1% SDS in dH_2_O. The resuspended samples were placed in Lysing Matrix B tubes (MPBio) and vortexed three times for 45 seconds, with cooling on ice between each vortexing interval. RNA was extracted using a phenol-chloroform protocol, while DNA was purified using the QIAmp DNA Mini kit. The quantification of 8-OHdG levels was conducted with the OxiSelect Oxidative DNA Damage ELISA kit (Cell Biolabs), and 8-OHG levels were assessed using the OxiSelect Oxidative RNA Damage ELISA kit (Cell Biolabs). Each sample was analyzed in six replicates.

### SEM analysis

Bacteria were cultured to an OD_600_ of 0.35, harvested by centrifugation, and washed with PBS. The PBS was then removed, and the cells were resuspended in 3% glutaraldehyde for fixation. Samples were subsequently washed three times with ultrapure water for 10 min. After 1 h to 2 h of fixation in 1% osmium tetroxide, they were washed again three times with ultrapure water for 10 min. Dehydration was performed using a graded series of ethanol solutions: 30%, 50%, 70%, 90%, and 100% (100% concentration repeated three times), with each step lasting 15 min. Samples were then dropped onto silicon wafers using a pipette and mounted onto a sample stage using conductive adhesive. The mounted samples were sputter-coated with gold using an ion sputtering instrument. Imaging was performed using a JSM-IT700HR scanning electron microscope (JEOL, Japan). Each sample was first observed at low magnification to assess its overall morphology, followed by high-resolution imaging of selected regions to identify specific bacterial morphological changes.

### *In vivo* murine infection experiments

Two experiments were conducted to evaluate the efficacy of TGC and Ado in reducing bacterial loads and improving survival in a murine infection model. A total of 24 male Balb/c mice (6 weeks old) received an intraperitoneal injection of 200 µL bacterial suspension at a concentration of 1 × 10^9^ CFU/mL. One hour post-infection, mice were randomly assigned to one of four treatment groups: physiological saline, TGC (10 mg/kg), Ado (70 mg/kg), or a combination therapy with both TGC and Ado. After a 24 h treatment period, all mice were euthanized, and their liver and spleen tissues were collected for analysis. Bacterial loads were determined by plate counting, while tissue samples underwent histopathological evaluation following fixation in formalin and staining with hematoxylin and eosin. In the second experiment, a total of 60 male Balb/c mice were infected with bacterial suspension and were randomly divided into four groups: a negative control group receiving physiological saline, and three treatment groups administered TGC (10 mg/kg), Ado (70 mg/kg), or combination therapy, respectively. Fifteen mice were injected with physiological saline as the negative control group. Survival rates for all groups were recorded at 24 h intervals.

### Histopathologic examination

Tissue samples from the liver and spleen of mice were fixed, dehydrated, and cleared. Subsequently, they were sectioned into 5 µm thick slices and mounted on glass slides. Initially, the tissue sections were heated to 40°C to ensure proper adhesion to the slides. Then, a graded ethanol dehydration series was performed, followed by clearing in xylene for enhanced transparency. Next, the sections were immersed in a staining rack containing hematoxylin and eosin dye solutions. The samples underwent hematoxylin immersion for 5–10 min to stain nuclei before being rinsed with tap water. They were then briefly dipped in acidic ethanol (1% hydrochloric acid in ethanol) for differentiation of staining and rinsed again. Eosin counterstaining was carried out for 2–5 min, followed by rinsing with tap water and subsequent dehydration. Finally, the stained samples were cleared using xylene, mounted with a coverslip using a permanent mounting medium, and allowed to dry completely prior to observation under an optical microscope for histological analysis.

### Biosafety procedures

In this study, we strictly adhered to Biosafety Level 2 (BSL-2) requirements in accordance with the guidelines from the Laboratory Biosafety Manual BSL-2 and BSL 2/3 and the Biosafety in Microbiological and Biomedical Laboratories. All experimental procedures were conducted in a laboratory equipped with a Class II biosafety cabinet. Laboratory personnel wore disposable nitrile gloves, lab coats, and goggles at all times, with N95 masks used when necessary. The handling of biological materials, emergency response to spills, and sterilization of waste by autoclaving were all performed following the procedures outlined in the aforementioned guidelines to ensure laboratory safety and experimental reliability. All personnel involved in the study received comprehensive biosafety training prior to the start of the experiments and were subject to regular supervision and assessment.

### Statistical analysis

Statistical significance was determined using unpaired two-sided Student’s *t*-tests or one-way analysis of variance. The levels of significance were as follows: *P  <  0.05, **P <  0.01.

## Data Availability

The original contributions presented in the study are included in the article/Supplementary Material, further inquiries can be directed to the corresponding author.

## SUPPLEMENTAL MATERIAL

Supplemental material spectrum.02382-24-s0001.pdf Fig. S1 and S2; Table S1.

ASM does not own the copyrights to Supplemental Material that may be linked to, or accessed through, an article. The authors have granted ASM a non-exclusive, world-wide license to publish the Supplemental Material files. Please contact the corresponding author directly for reuse.
