# Supplementary material for: Exogenous adenosine counteracts tigecycline resistance in tet(X3)-harboring Escherichia coli
Source: Microbiol Spectr. 2025 Jul 7;13(8):e02382-24. doi: 10.1128/spectrum.02382-24 (PMC12323663; doi:10.1128/spectrum.02382-24)
Supplement: Supplemental material — Fig. S1 and S2; Table S1. [file spectrum.02382-24-s0001.pdf]

Supplemental Information for

Exogenous adenosine reverses tigecycline resistance in *tet*(X3)-carrying *Escherichia coli*

Jing Sun, Yiming Liu, Jiashen Chang, Ying Liu, Luqi Li, Ran Jiang, Yihan Luo, Shuo Yang, Mei Yang, Xinglong Wang, Juan Wang, Xi Xia, Kangkang Guo, Zengqi Yang, Dongyang Ye

Table of Content

Figure S1……………………………………………………………………**S3**

Figure S2……………………………………………………………………**S4**

Table S1………………………………………………………………………**S5**

(A)

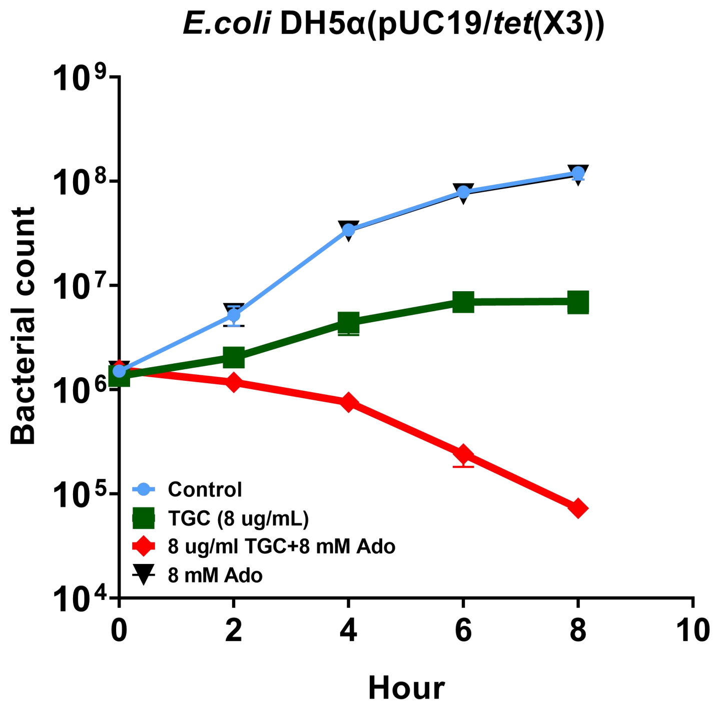
E.coli DH5α(pUC19/tet(X3))
Bacterial count
10^9
10^8
10^7
10^6
10^5
10^4
0
2
4
6
8
10
Hour
Control
TGC (8 ug/mL)
8 ug/ml TGC+8 mM Ado
8 mM Ado

(B)

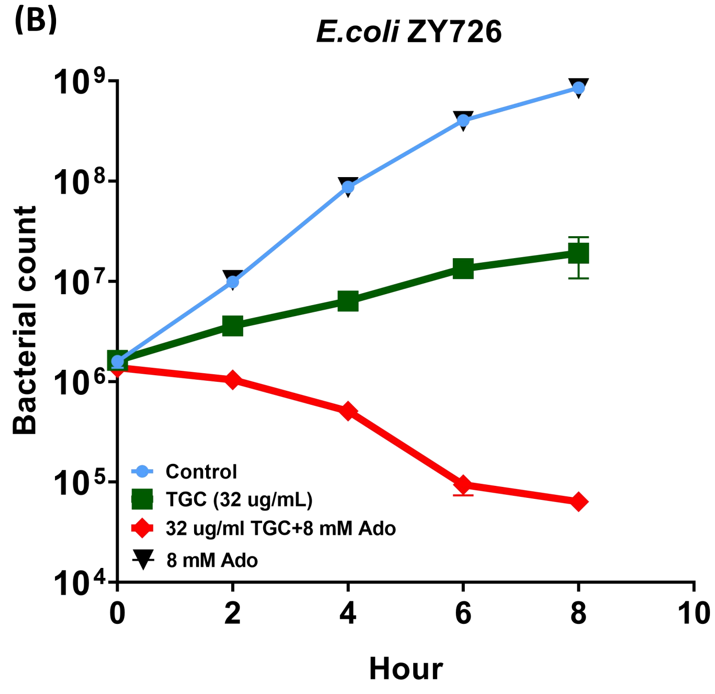
E.coli ZY726
Bacterial count
10^9
10^8
10^7
10^6
10^5
10^4
0
2
4
6
8
10
Hour
Control
TGC (32 ug/mL)
32 ug/ml TGC+8 mM Ado
8 mM Ado

(C)

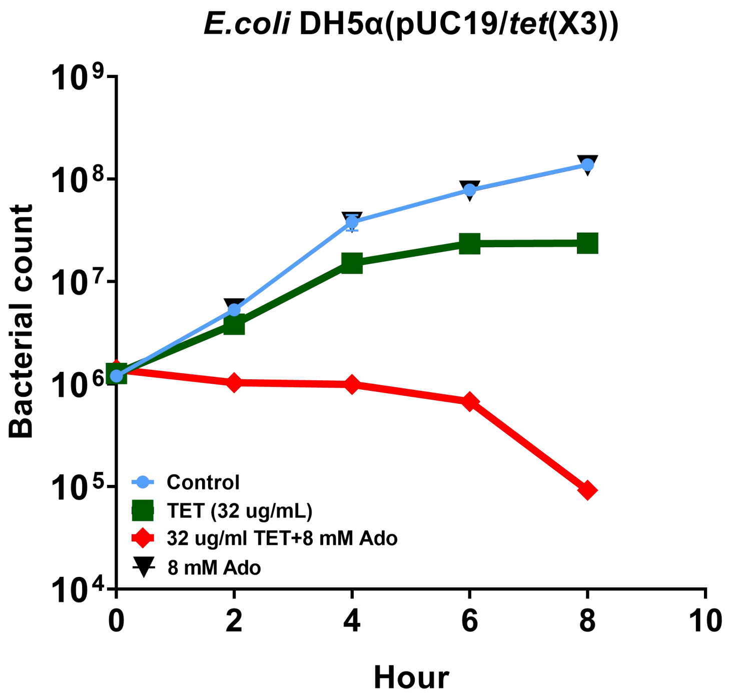

(D)

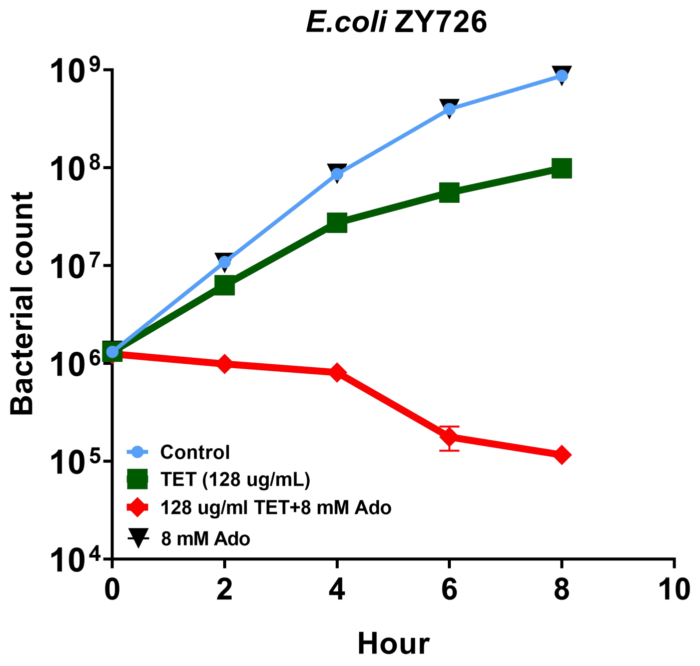

Figure S1. Growth curve of wild-type and engineered strains following exposure to tigecycline, tetracycline, adenosine, or their combination.

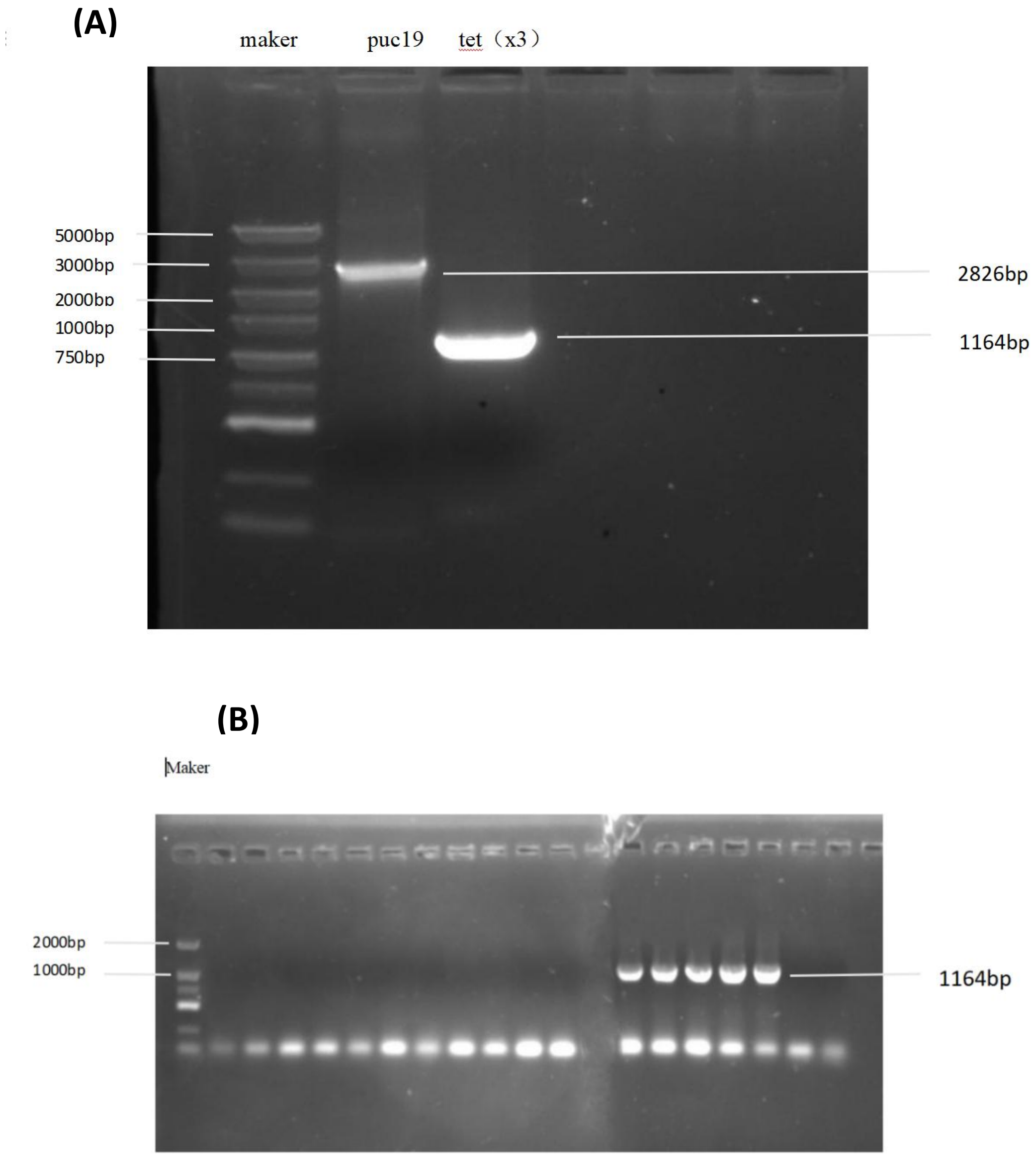

Figure S2. Construction of the engineering strain. (A) PCR amplification of the *tet*(X3) gene and vector pUC19. (B) PCR identification of the cloned vector pUC19/*tet*(X3).

Table S1 Metabolic regulatory alterations in *tet*(X3)-positive *E. coli*

Compound	Mode	Adducts	Formula	Compound ID	*P*-value	Fold Change
**Upregulated**						
Pantothenate	Amide NEG	M-H	$C_9H_{17}NO_5$	C00864	0.000122503	8.820291002
Tartaric acid	Amide NEG	M-H	$C_4H_6O_6$	C00552	0.000412379	44.33781497
2',3'-Cyclic GMP	Amide NEG	M-H	$C_{10}H_{12}N_5O_7P$	C06194	0.000606598	4.944629847
Adenosine-5'-pentaphosphate	Amide NEG	M-H	$C_{10}H_{18}N_5O_{19}P_5$	C20198	0.000904037	6.433095468
ADP	Amide NEG	M-H	$C_{10}H_{15}N_5O_{10}P_2$	C00008	0.00102779	8.95985394
ADPglucose	Amide NEG	M-H	$C_{16}H_{25}N_5O_{15}P_2$	C00498	0.00057652	8.034253969
Cinnamic acid	Amide NEG	M-H	$C_9H_8O_2$	C00423	0.000410543	2.808393894
Citrate	C_{18} POS	M+H	$C_6H_5O_7$	C00158	0.00038241	2.488321385
Cytosine	Amide POS	M+H	$C_4H_5N_3O$	C00380	0.000311781	14.59262945
Deoxycholic acid	C_{18} POS	M+H	$C_{24}H_{40}O_4$	C04483	0.009633091	2.278106395
Deoxyuridine	Amide NEG	M-H	$C_9H_{12}N_2O_5$	C00526	0.000400819	5.121640827
Gluconic acid	Amide NEG	M-H	$C_6H_{12}O_7$	C00257	9.4183E-05	70.3919258
Glucose	Amide NEG	M-H	$C_6H_{12}O_6$	C00031	0.000305387	16.78765395
Glucuronate	Amide POS	M+H	$C_6H_9O_7$	C00191	0.001603404	4.629985764
Dihydrothymine	Amide POS	M+H	$C_5H_8N_2O_2$	C00906	0.015064784	1.659014854
Dihydrouracil	Amide POS	M+H	$C_4H_6N_2O_2$	C00429	0.001428005	2.653155703
Folic acid	Amide NEG	M-H	$C_{19}H_{19}N_7O_6$	C00504	8.4637E-05	13.82440844
Folinic acid	Amide NEG	M-H	$C_{20}H_{23}N_7O_7$	C03479	0.001128581	2.723227026
Glucuronic acid	C_{18} NEG	M-H	$C_6H_{10}O_7$	C00191	0.003457404	2.68733502
Isocitrate	C_{18} POS	M+H	$C_6H_5O_7$	C00311	0.001703342	3.490681358
Lactose	Amide NEG	M-H	$C_{12}H_{22}O_{11}$	C00243	1.89383E-06	3.872518883
Ascorbate	C_{18} NEG	M-H	$C_6H_7O_6$	C00072	0.005147074	2.074807

Glutamate	Amide POS	M+H	$C_5H_7NO_4$	C00025	0.000682856	14.99603022
Glutamate 5-phosphate	Amide NEG	M-H	$C_5H_{10}NO_7P$	C03287	0.010941081	2.058930285
Homocysteine	C_{18} NEG	M-H	$C_4H_9NO_2S$	C00155	5.55596E-05	9.253567217
Lysine	Amide NEG	M-H	$C_6H_{14}N_2O_2$	C00047	9.18646E-05	6.757745011
NMNH	Amide NEG	M-H	$C_{11}H_{17}N_2O_8P$	C21113	0.002451928	2.422339846
Oxalyl-CoA	Amide NEG	M-H	$C_{23}H_{36}N_7O_{19}P_3S$	C00313	1.67422E-05	10.31281077
Phosphonoacetate	C_{18} POS	M+H	$C_2H_5O_5P$	C05682	0.000166003	2.814735818
Pyridoxal	Amide POS	M+H	$C_8H_9NO_3$	C00250	0.000391173	12.30773769
Pyridoxamine	C_{18} POS	M+H	$C_8H_{12}N_2O_2$	C00534	2.84282E-05	12.526843
Pyridoxine 5'-phosphate	Amide NEG	M-H	$C_8H_{12}NO_6P$	C00627	0.001956939	1.824828628
Salicylic acid	Amide NEG	M-H	$C_7H_6O_3$	C00805	0.000244958	71.69871812
Tetrahydrofolic acid	Amide NEG	M-H	$C_{19}H_{23}N_7O_6$	C00101	7.09476E-05	6.691791281
Ubiquinone-8	C_{18} NEG	M-H	$C_{49}H_{74}O_4$	C17569	0.000792916	4.261740169
**Downregulated**						
Pantetheine	Amide NEG	M-H	$C_{11}H_{22}N_2O_4S$	C00831	0.000837867	39.63554794
Adenine	Amide NEG	M-H	$C_5H_5N_5$	C00147	0.001155709	5.707205193
Adenosine	Amide NEG	M-H	$C_{10}H_{13}N_5O_4$	C00212	0.000913978	6.587997902
cAMP	Amide NEG	M-H	$C_{10}H_{12}N_5O_6P$	C00575	0.000169206	24.43251058
Chitobiose	Amide POS	M+H	$C_{16}H_{28}N_2O_{11}$	C01674	0.000247352	8.292907235
cis-2-Methylaconitate	Amide NEG	M-H	$C_7H_8O_6$	C04225	0.000741069	8.18376878
Cytidine	Amide NEG	M-H	$C_9H_{13}N_3O_5$	C00475	0.000205254	6.659518116
Xylose	C_{18} POS	M+H	$C_5H_{10}O_5$	C00181	0.000263142	25.17588181
Fumarate	C_{18} NEG	M-H	$C_4H_2O_4$	C00122	0.00137697	2.188963745
Galactitol	C_{18} POS	M+H	$C_6H_{14}O_6$	C01697	0.000221036	6.212478336
Gallic acid	Amide NEG	M-H	$C_7H_6O_5$	C01424	0.000562286	10.19037772
Guanosine	Amide NEG	M-H	$C_{10}H_{13}N_5O_5$	C00387	0.006727499	1.979172835

Heptadecenoic acid	C_{18} NEG	M-H	$C_{17}H_{32}O_2$	C16536	0.000360833	10.1548071
Hypoxanthine	Amide POS	M+H	$C_5H_4N_4O$	C00262	0.001313061	5.019495866
Glutamic acid	Amide NEG	M-H	$C_5H_9NO_4$	C00025	0.000284677	8.017906051
Arginine	C_{18} POS	M+H	$C_6H_{14}N_4O_2$	C00062	1.87717E-06	2.94665747
Aspartate	C_{18} POS	M+H	$C_4H_5NO_4$	C00049	0.004739823	2.139853779
Cystathionine	C_{18} POS	M+H	$C_7H_{14}N_2O_4S$	C02291	1.6102E-05	22.70153911
Lipoamide	Amide NEG	M-H	$C_8H_{15}NOS_2$	C00248	8.20405E-05	14.42277043
Leucine	C_{18} NEG	M-H	$C_6H_{13}NO_2$	C00123	0.000675169	1.792264836
Pyroglutamic acid	Amide NEG	M-H	$C_5H_7NO_3$	C01879	3.88189E-05	39.77148569
Valine	Amide POS	M+H	$C_5H_{11}NO_2$	C00183	0.001113348	11.54009016
Menadiol	C_{18} POS	M+H	$C_{11}H_{10}O_2$	C07126	0.001801978	2.141744874
Menaquinone	Amide NEG	M-H	$C_{51}H_{72}O_2$	C00828	0.00033606	65.55269732
Nicotinamide adenine dinucleotide	Amide POS	M+H	$C_{21}H_{27}N_7O_{14}P_2$	C00003	0.000198381	26.32290593
NMN	Amide NEG	M-H	$C_{11}H_{15}N_2O_8P$	C00455	0.000466358	2.764394089
o-Succinylbenzoate	C_{18} POS	M+H	$C_{11}H_{10}O_5$	C02730	0.000193005	28.08457847
S-Lactoylglutathione	Amide POS	M+H	$C_{13}H_{21}N_3O_8S$	C03451	5.65667E-05	53.74414705
Succinic acid	Amide NEG	M-H	$C_4H_6O_4$	C00042	0.000302776	10.56333018
UDP-N-acetylmuramate	C_{18} NEG	M-H	$C_{20}H_{28}N_3O_{19}P_2$	C01050	0.000696089	28.31689292
Undecaprenyl phosphate	Amide NEG	M-H	$C_{55}H_{91}O_4P$	C17556	0.000177473	9.728568687
Uracil	Amide NEG	M-H	$C_4H_4N_2O_2$	C00106	4.24719E-05	5.843331478
Uric Acid	Amide NEG	M-H	$C_5H_4N_4O_3$	C00366	0.000151282	5.563026182
Urocanic acid	C_{18} POS	M+H	$C_6H_6N_2O_2$	C00785	0.003075461	2.142076923
Xanthine	Amide NEG	M-H	$C_5H_4N_4O_2$	C00385	0.004888389	2.524090495
Xanthosine	Amide NEG	M-H	$C_{10}H_{12}N_4O_6$	C01762	0.000409281	4.132685207
α-Linolenic acid	C_{18} POS	M+H	$C_{18}H_{30}O_2$	C06427	4.04469E-05	2.410326438
